# A Novel Role of RASSF9 in Maintaining Epidermal Homeostasis

**DOI:** 10.1371/journal.pone.0017867

**Published:** 2011-03-21

**Authors:** Chiou-Mei Lee, Polung Yang, Lih-Chyang Chen, Chia-Chun Chen, Shinn-Chih Wu, Hsiao-Yun Cheng, Yu-Sun Chang

**Affiliations:** 1 Department of Medical Research and Development, Chang Gung Memorial Hospital at Lin-Kou, Taoyuan, Taiwan; 2 Graduate Institutes of Biomedical Sciences, Chang Gung University, Taoyuan, Taiwan; 3 Chang Gung Molecular Medicine Research Center, Chang Gung University, Taoyuan, Taiwan; 4 Department of Animal Science and Technology, National Taiwan University, Taipei, Taiwan; University of Western Ontario, Canada

## Abstract

The physiological role of RASSF9, a member of the Ras-association domain family (RASSF), is currently unclear. Here, we report a mouse line in which an Epstein-Barr virus Latent Membrane Protein 1 (*LMP1*) transgene insertion has created a 7.2-kb chromosomal deletion, which abolished *RASSF9* gene expression. The *RASSF9*-null mice exhibited interesting phenotypes that resembled human ageing, including growth retardation, short lifespan, less subcutaneous adipose layer and alopecia. In the wild-type mice, RASSF9 is predominantly expressed in the epidermal keratinocytes of skin, as determined by quantitative reverse-transcription PCR, immunofluorescence and *in situ* hybridization. In contrast, *RASSF9*−/− mice presented a dramatic change in epithelial organization of skin with increased proliferation and aberrant differentiation as detected by bromodeoxyuridine incorporation assays and immunofluorescence analyses. Furthermore, characteristic functions of *RASSF9*−/− versus wild type (WT) mouse primary keratinocytes showed significant proliferation linked to a reduction of p21Cip1 expression under growth or early differentiation conditions. Additionally, in *RASSF9*−/− keratinocytes there was a drastic down-modulation of terminal differentiation markers, which could be rescued by infection with a recombinant adenovirus, Adv/HA-*RASSF9*. Our results indicate a novel and significant role of RASSF9 in epidermal homeostasis.

## Introduction

The RASSF proteins comprise an evolutionarily conserved protein family of ten members (RASSF1 to RASSF10) in vertebrates [1]. RASSF1 through RASSF6 all harbor C-terminal Ras-association (RA) domains and are grouped together as the C-terminal RASSF family, while RASSF7 through RASSF10 all contain N-terminal RA domains and are grouped as the N-terminal RASSF family. The members of the N-terminal RASSF family lack the characteristic Sav–RASSF–Hpo (SARAH) domains found in members of the C-terminal RASSF family [2]. The C-terminal RASSFs, as well as RASSF8 and RASSF10 of the N-terminal RASSFs, have been reported to show reduced expression in various cancers, and are therefore considered to be tumor suppressors [2], [3], [4], [5]. RASSF9 was originally named P-CIP1 (PAM C-terminal interactor 1) following its identification as a peptidylglycine-amidating monooxygenase (PAM)-trafficking protein, which has been linked to the recycling of endosomes [6] and interaction with N-, K- and R-Ras proteins in GST pull-down assays [7]. Although the gene encoding *RASSF9* is known to be expressed in multiple organs, including testis, kidney, skeletal muscle, liver, lung, brain, and heart [6], its biological and physiological roles are not yet fully understood.

Recently, we obtained an Epstein-Barr virus Latent Membrane Protein 1 (*LMP1*) transgene-insertion-derived mutant mouse line, which was a *RASSF9* deficient mutant with phenotype typical of the alopecia syndrome. In normal skin, the proliferative cells are confined to a single basal layer, and the non-proliferative differentiating cells are located in the suprabasal layers. At the final stage of differentiation, the stratum corneum is formed at the outer layer of the epidermis, where it serves as a barrier that prevents epidermal water loss [8]. The stratum corneum is composed of a number of proteins, including loricrin, involucrin and filaggrin, all of which are associated with keratin intermediate filaments [9]. Calcium-induced differentiation of primary mouse keratinocytes in culture provides a well-established model for the complex program of differentiation that occurs *in vivo* in the transition from the basal to upper epidermal layers [10]. Since a particular group of epithelial cells among epidermal epithelium is responsible for generating new hair follicle epithelium during each hair cycle [11], [12], the intriguing phenotype of the mutant mice therefore prompted us to investigate the possibility that RASSF9 plays some important roles in regulating epidermal homeostasis.

Here, we show that RASSF9 is predominantly expressed in epidermal keratinocytes of skin, and loss of RASSF9 expression results in hyperplasia and aberrant differentiation of epidermis. The results of our *in vitro* study of mouse primary keratinocytes showed that RASSF9 mediated growth suppression and activation of the differentiation program. The mechanism by which RASSF9 mediates keratinocyte growth suppression may rely on the regulation of cell-cycle inhibitor p21Cip1, as demonstrated by the results of reciprocal alterations between deficient RASSF9 expression and its compensation in mouse primary keratinocytes. Taken together, our findings show that RASSF9 is essential for the maintenance of epidermal homeostasis.

## Materials and Methods

### Animals

All animal experiments were approved by the Institutional Animal Care and Use Committee (IACUC) at the Chang Gung University, Taiwan (Permit Number: CGU10-027), and were carried out in accordance with the relevant guidelines. The mice were bred and maintained under a consistent temperature of 21–23°C, a relative humidity of 50–70%, and a 12-hr light-dark cycle with normal access to water and chow. In all experiments, mice were sacrificed by ether inhalation.

### Generation of transgenic mice

The utilized transgene was the nasopharyngeal carcinoma (NPC)-derived *LMP1* (*NLMP1*) gene, including the upstream promoter, coding sequences, and the 5′-untranslated region [13]. Transgenic mice were generated as follows. Briefly, the relevant *Eco*RI-*Hind*III fragment was injected into fertilized oocytes from ICR strain mice. Founders were crossed with ICR mice, and PCR and Southern blot analysis were used to screen progeny for the presence of the transgene.

### Southern blotting

For Southern blotting, 20 µg of tail-tissue-derived genomic DNA was digested with either *EcoR*I or *Hind*III, which do not cut the transgene. The digests were resolved by electrophoresis, and the DNA was transferred to a nylon membrane (Hybond-N, Amersham Biosciences, Piscataway, NJ). Probes were prepared using the PCR DIG Probe Synthesis Kit (Roche Molecular Biochemicals, Mannheim, Germany). The probes were designed from the transgene sequence or the mutated genome, and were designated Tg_probe or Δ_probe, respectively (Table S1). Southern blotting was performed according the DIG system's protocol for filter hybridization (Roche Molecular Biochemicals, Mannheim, Germany).

### Inverse PCR amplification of the transgene-flanking genomic sequences

Tail-derived genomic DNA was completely digested with *Taq*I or *Bcl*I, and then self-circularized with T4 DNA ligase (2∼5 µg/mL). The ligation mixture was extracted with phenol:chloroform (1∶1,v/v) followed by chloroform alone, and DNA was precipitated with ethanol and sodium acetate (pH 5.2). The self-ligated DNA (1 µg) was used as the template for PCR amplification of the *Eco*RI and *Hind*III flanking genome sequence of the insertion fragment. The utilized primers were specific for both end sequences of the transgenic DNA (Supplementary Information Table S1).

### RNA isolation

RNA from tissues and keratinocytes was prepared by TRIzol extraction according to manufacturer's protocol (Invitrogen, Karlsruhe, Germany).

### RT-PCR and QRT-PCR

One µg of total RNA was subjected to reverse transcription using an oligo-dT primer (Invitrogen, Carlsbad, CA). The reaction mixture was then amplified using gene-specific intron-crossing oligonucleotide primer pairs specific to the genes encoding mouse *RASSF9*, *p21Cip1* and the reference genes of β-Actin, 3-phosphate dehydrogenase (*GAPDH*) and hypoxanthine phosphoribosyltransferase (*HPRT1*). Quantitative RT-PCR was performed on a LightCycler (Roche Molecular Biochemicals, Mannheim, Germany) according to manufacturer's instructions, using the FastStart DNA Master SYBR Green I mix (Roche Molecular Biochemicals, Mannheim, Germany). The amplification conditions were as follows: 95°C for 10 minutes followed by 45 cycles of 95°C for 10 seconds, 60°C for 10 seconds, and 72°C for 10 seconds. The cycle threshold (Ct) values were normalized with respect to those of the reference genes. The oligonucleotide sequences of the utilized primers are listed in Supplementary Information Table S1.

### BrdU-incorporation assays *in vivo* and *in vitro*

Mice were *in vivo* pulse-labeled with BrdU (250 µg/g body weight; Sigma Chemical Corp., St Louis, MO) via intraperitoneal (i.p.) injection, and skin tissues were harvested after two-hr incubation. Each sample was fixed in formalin, embedded in paraffin, and sectioned at 4 µm. The sections were deparaffinized, rehydrated, subjected to antigen retrieval in citrate buffer, and then subjected to immunohistochemical staining using specific antibodies against BrdU (Serotec, Oxford, UK) and keratin 14 (Covance, Berkeley, CA) according to the protocol for the utilized anti-bromodeoxyuridine-fluorescein kit (Roche Molecular Biochemicals, Mannheim, Germany), with the DNA-denaturation step modified to incubating the slides in 2 M HCl, for 20 min at 37°C. Chicken anti-rat IgG conjugated with FITC was used for BrdU detection (Santa Cruz, CA), while goat anti-rabbit IgG conjugated with TRITC was used for K14 detection (Jackson ImmunoResearch Laboratories, West Grove, PA). Nuclei were stained with 4′6-diamidin-2-phenylindol-dihydrochloride (DAPI; Sigma Chemical Corp., St Louis, MO). The resulting images were examined and photographed under a laser-scanning confocal microscope (Leica TCS SP2; Leica, Germany or LSM 510 Meta NLO; Carl Zeiss, Inc., USA).

For the *in vitro* assay, mouse primary keratinocytes were seeded to 48-well plates and incubated with growth medium (0.06 mM calcium) for 17 hr, followed by infection with or without a RASSF9-encoding recombinant adenovirus for 24 hr. Subsequently, the cultures were plated with fresh medium containing 0.06 mM calcium and incubated for another 48 hr. Keratinocytes were pulse-labeled with 10 µM BrdU for 2 hr and then harvested, and BrdU incorporation was analyzed by ELISA according to the manufacturer's protocol (Cell Proliferation Biotrak ELISA System; Amersham Biosciences, Piscataway, NJ). The BrdU immunofluorescence staining was performed according to the protocol described above.

### Bacterial expression of RASSF9, and anti-RASSF9 antibody preparation

The 285-1625nt fragment of the mouse *RASSF9* cDNA (NCBI RefSeq NM_146240) was cloned into the *Eco*RI-*Xho*I sites of the pGEX-4T-1 vector (Amersham Biosciences, Piscataway, NJ). The resulting pGEX-4T-1-*RASSF9* plasmids were transformed into *Escherichia coli* BL21 competent cells and grown to the appropriate cell density at 37°C with Ampicillin selection. Protein expression was induced by addition of 1 mM isopropyl-1-thio-β-D-galactopyranoside (IPTG) for 4 hr at 37°C, and the resulting GST-RASSF9 fusion protein with a GST tag in the N-terminus were purified and used to generate antiserum in rabbits. Specificity of anti-RASSF9 antibody is confirmed by Western immunoblotting of exogenously expressed RASSF9 protein (Figure S1).

### Histology and immunofluorescence

Dorsal skin of mouse was fixed in 10% neutral buffered formalin, embedded in paraffin, and sectioned at 4 µm. The sections were deparaffinized, rehydrated, and then subjected to H&E staining or Masson's trichrome staining. The resulting images were examined and photographed under a microscope (Axioplan II; Carl Zeiss Microimaging Inc., Germany). For immunofluorescence analysis, OCT-embedding frozen skin was cut into 4-µm sections, fixed in ice-cold acetone for 30 sec (for RASSF9 immunofluorescence staining) or neutral buffered formalin for 15 min, and subjected to double-indirect immunostaining according to the IHC World protocol (http://www.ihcworld.com/protocols/general_IHC). Finally, the specimens were mounted with Vectashield (Vector Labs, Burlingame, CA) and photographed by laser-scanning confocal microscopy as earlier described. The primary antibodies used in these experiments were as follows: anti-keratin 1, keratin 5, keratin 6, keratin 10 and keratin 14 polyclonal antibodies (Covance, Berkeley, CA); rabbit anti-RASSF9 (generation described in Materials and Methods). The utilized secondary antibodies were FITC- or TRITC-conjugated (Jackson ImmunoResearch Laboratories, West Grove, PA). Nuclei were stained with DAPI.

### Construction of the recombinant adenovirus

The full-length *RASSF9* gene was obtained from the RNA of normal ICR mouse brain using RT-PCR. The resulting 1.3-kb fragment was cloned into pCMV-HA (Clontech, Palo Alto, CA) and amplified using specific primers designed to generate an HA-tagged *RASSF9* fragment. The amplified fragment was subcloned into the *Not*I-*Xho*I sites of pShuttle-CMV and inserted into the adenoviral backbone plasmid, pAdEasy-1, for amplification in bacteria. Recombinant adenoviruses containing the *RASSF9* gene were generated according to the protocol for the utilized adenovirus-construction system (http://www.coloncancer.org/adeasy/protocol.htm). A GFP-encoding recombinant adenovirus was used as the control. Expression of RASSF9 in adenovirally infected keratinocytes was confirmed by immunoblotting with anti-HA antibody. Titration of recombinant adenovirus was performed by a plaque forming unit (PFU) assay in 293 cells following the manufacturer's instructions (Cell Biolabs).

### Cell culture and viral infection

The isolations and incubations of epidermal keratinocytes were done according to a web-published protocol (http://openwetware.org/wiki/Mouse_keratinocyte_cultures) with minor modifications. Briefly, epidermis was isolated from the skin of neonatal mice by incubation with Dispase II overnight at 4°C. Epidermal keratinocytes were dissociated by incubation of the skin samples in Trypsin-EDTA for 20 min at room temperature, and the cells were harvested by passing each sample through a sterile nylon 100-µm cell strainer (BD Falcon, Bedford, MA). The epidermal keratinocytes were then centrifuged, resuspended, plated onto collagen pre-coated dishes, and cultured in Eagle's minimal essential medium (EMEM; Cambrex, East Rutherford, NJ) supplemented with 4% Chelex-100 treated fetal bovine serum (FBS) (Bio-Rad Laboratories, Hercules, CA), 0.4 µg/mL hydrocortisone, 5 µg/mL insulin, 10 ng/mL epidermal growth factor (EGF), 10^−10^ M cholera toxin, 2×10^−9^ M triiodothyronine (T3), 100 units/mL penicillin, 100 µg/mL streptomycin, 2 mM L-Glu, and 0.06 mM CaCl_2_. After 17 hr, the culture medium was refreshed, and the cells were exposed to the recombinant adenovirus. The inoculating medium was removed 24 hr post infection. After infection, the cells were incubated in fresh media containing 0.06 mM or 2 mM calcium for the indicated time. Recombinant adenoviruses were produced and routinely used at a multiplicity of infection (MOI) of 10, unless otherwise specified.

### Western blotting

Adherent primary cells were harvested and homogenized at 4°C for 20 min in RIPA lysis buffer (25 mM TRIS, pH 7.5, 150 mM NaCl, 1% NP40, 1% Na-deoxycholate, and 0.1% SDS) containing 1 mM phenylmethylsulfonyl fluoride (PMSF), 0.3 µM aprotinin, 130 µM bestatin, 1 mM EDTA, and 1 mM leupeptin (Sigma Chemical Corp., St Louis, MO). Each lysate was cleared of cell debris by centrifugation, and each sample's total protein concentration was determined using the Bradford protein assay reagent (Bio-Rad, Hercules, CA). Equal amounts of protein were resolved by electrophoresis on SDS-polyacrylamide gels (Bio-Rad, Hercules, CA), and then transferred to a polyvinylidene difluoride membrane (PVDF) (Millipore Corp., Bedford, MA). Immunoblotting was performed as previously described [14] using the following specific primary antibodies: anti-filaggrin, anti-loricrin, anti-keratin 14 (Covance, Berkeley, CA); anti-HA (Sigma Chemical Corp., St Louis, MO) and anti-p21Cip1 (F-5; Santa Cruz, CA). The proteins of interest were reacted with a horseradish peroxidase-conjugated secondary antibody, and then detected with an enhanced chemiluminescence system (Amersham Biosciences, Piscataway, NJ). K14, which is constitutively expressed in keratinocytes irrespective of the cell-growth conditions, was used as the loading control.

### *In situ* hybridization

Fresh dorsal skin tissue was embedded in optimum cutting temperature (OCT) compound (Tissue-Tek, Sakura Finetek, Torrance, CA), and 4-µm cryosections were cut and subjected to *in situ* hybridization according to the protocols of the Anderson lab (California Institute of Technology, Pasadena, CA; http://wmc.rodentia.com/docs/Big_In_Situ.html). Fragments corresponding to nucleotides (nt) 285–817 and 1126–1625 of the mouse *RASSF9* cDNA (NCBI RefSeq NM_146240) were separately cloned into the *Eco*RI-*Xho*I sites of the pBluescript II KS (+) phagemid vector (Stratagene, La Jolla, CA) for riboprobe synthesis. Digoxigenin-UTP (DIG)-labeled antisense and sense riboprobes were prepared by *in vitro* transcription with DIG-11-UTP and either T3 or T7 RNA polymerase, according to the manufacturer's protocol (DIG Application Manual for In Situ Hybridization, 3^rd^ edition; Roche Molecular Biochemicals, Mannheim, Germany). The hybridization signal was detected as a dark precipitate that formed following exposure to an alkaline phosphatase-labeled anti-DIG antibody and a substrate containing nitroblue tetrazolium (NBT) (Promega, Madison, WI) and 5-bromo-4-chloro-3-indolylphosphate (BCIP) (Promega, Madison, WI).

### Affymetrix Analysis

Total RNA was extracted from mouse primary keratinocytes of the neonatal WT (n = 1) and *RASSF9*−/− mice (n = 2) by TRIzol extraction per manufacturer's instructions(Invitrogen). Briefly, one 10-cm culture plate of confluent monolayer of primary keratinocytes per sample was lysed in 1 mL TRIzol reagent after maintenance for two days in low-calcium keratinocyte serum-free medium with supplements (Invitrogen, Carlsbad, CA). The cell lysate was mixed with 0.2 mL of chloroform and centrifuged at 12,000× g for 10 minutes at 4°C. Total RNA was precipitated from the resultant aqueous phase by isopropanol, washed in 70% v/v ethanol, air-dried and then resuspended in RNAse-free water for subsequent assays. The A260/A280 ratio of all RNA samples was between 1.8 and 2.1, and sample quality was verified by electrophoresis on 1% agarose gel for 18S and 28S ribosomal RNAs. The resultant samples of total RNA were submitted to the Microarray & Gene Expression Analysis Core Facility, VGH National Yang-Ming University Genome Research Center, Taipei, Taiwan, for subsequent processing and assay with Affymetrix GeneChip Mouse Genome 430 2.0 Array (Affymetrix, Fremont, CA). All the data is MIAME compliant, and the raw datasets are deposited in MIAME-compliant NCBI-Gene Expression Omnibus (GEO) database (Accession Number: GSE24190). The resulting dataset was analyzed using GeneSpring software (Silicon Genetics, Agilent Technologies, Santa Clara, CA).

### Image quantitation and statistical analysis

Quantitation of image data was achieved using the image-analysis programs ImageJ. (version 1.41) and ImageQuant (version 5.1). Analysis for statistical significance was examined using t-tests. *P* values <0.05 were considered statistically significant.

## Results

### Characteristics of a transgene-insertion-mutation mouse line

**Figure 1:**
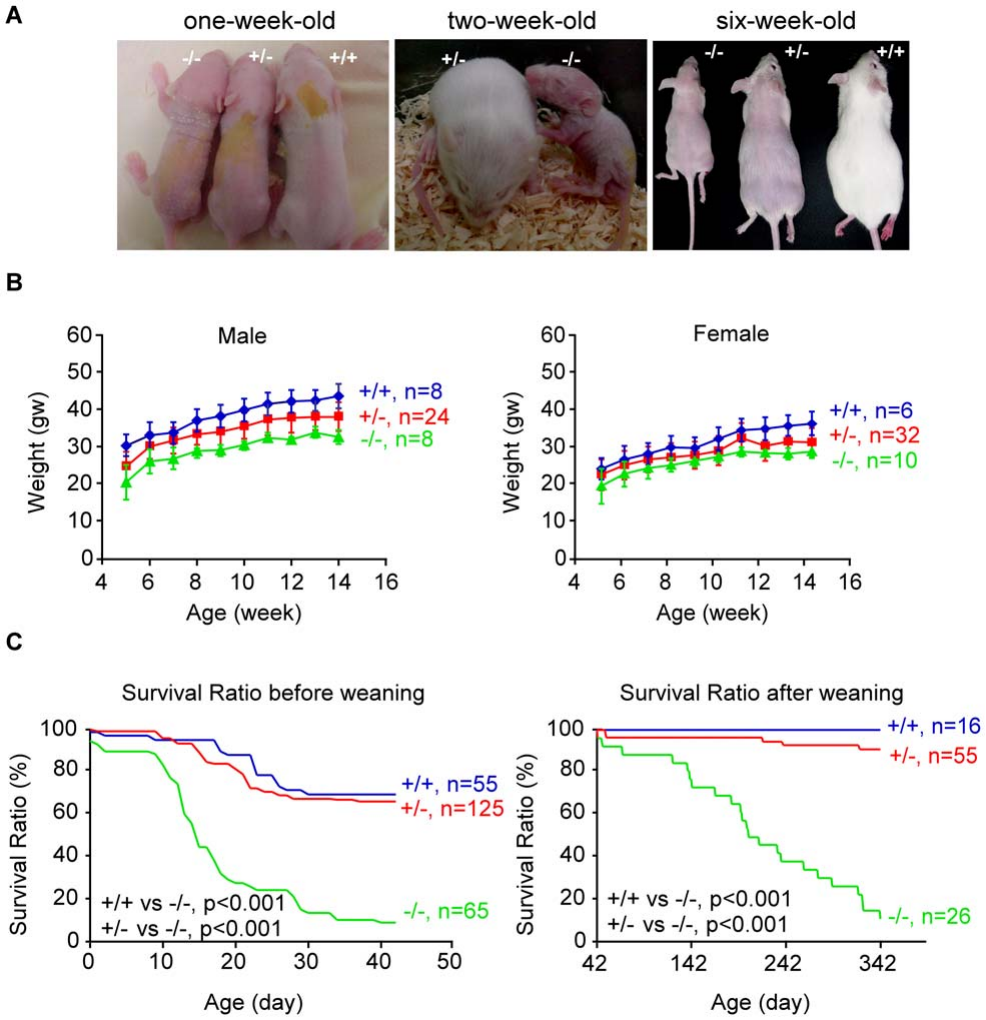
Characterization of the transgenic mutant mouse line. (A) Macroscopic phenotypes of mice of various genotypes were shown at one, two, and six weeks after birth. (B) Growth curve analysis. The growth curves of the different genotypes exhibited an effect of haploinsufficiency on body weight. The body weight of the homozygous mutant was severely reduced as compared to that of the heterozygous and WT mice. (C) The survival rate. The survival rate of transgenic mutant mice was lower than that of the WT mice. Statistical analyses were performed using the SPSS13.0 software. *P*<0.05. +/+, wild type; +/−, heterozygous mutant; −/−, homozygous mutant.

Out of 21 independent Epstein-Barr virus (EBV) latent membrane protein 1 (*LMP1*) transgenic mice, one mouse line exhibited intriguing phenotypes showing growth retardation, alopecia and short lifespan in homozygotes (−/−; Figure 1A–C), and signs of haploinsufficiency for heterozygotes (+/−) for the alopecia (Figure 1A). Homozygous mice (−/−) were born with normal Mendelian frequency but started to die off rapidly after two weeks past birth. At six weeks past birth, approximately 10% of homozygous mutants survived to wean (Figure 1C, left panel), but no remaining homozygotes survived beyond one year after weaning. Overall, homozygous exhibited severe growth retardation and short lifespan compared to heterozygous and the WT mice (Figure 1A–C). The mRNA transcript of *LMP-*1 transgene in this line was not efficiently expressed in the neonate skin at the onset of the pathology (Figure S2A, top panel). Meanwhile, LMP-1 protein expression in the skin of the transgenic mice at two weeks of age was not detectable by Western blotting with anti-LMP-1 antibody (Figure S2B). Thus, it is likely that the observed skin pathology of the transgenic mice may be attributable to abolition of endogenous gene expression rather than the consequence of transgenic LMP-1 expression. Taken together, the results strongly suggest that the transgene insertion in this mutant line unexpectedly disrupted a gene whose expression was crucial for development.

### Chromosomal deletion and gene silencing of the *RASSF9* gene in the homozygous mutant line

**Figure 2:**
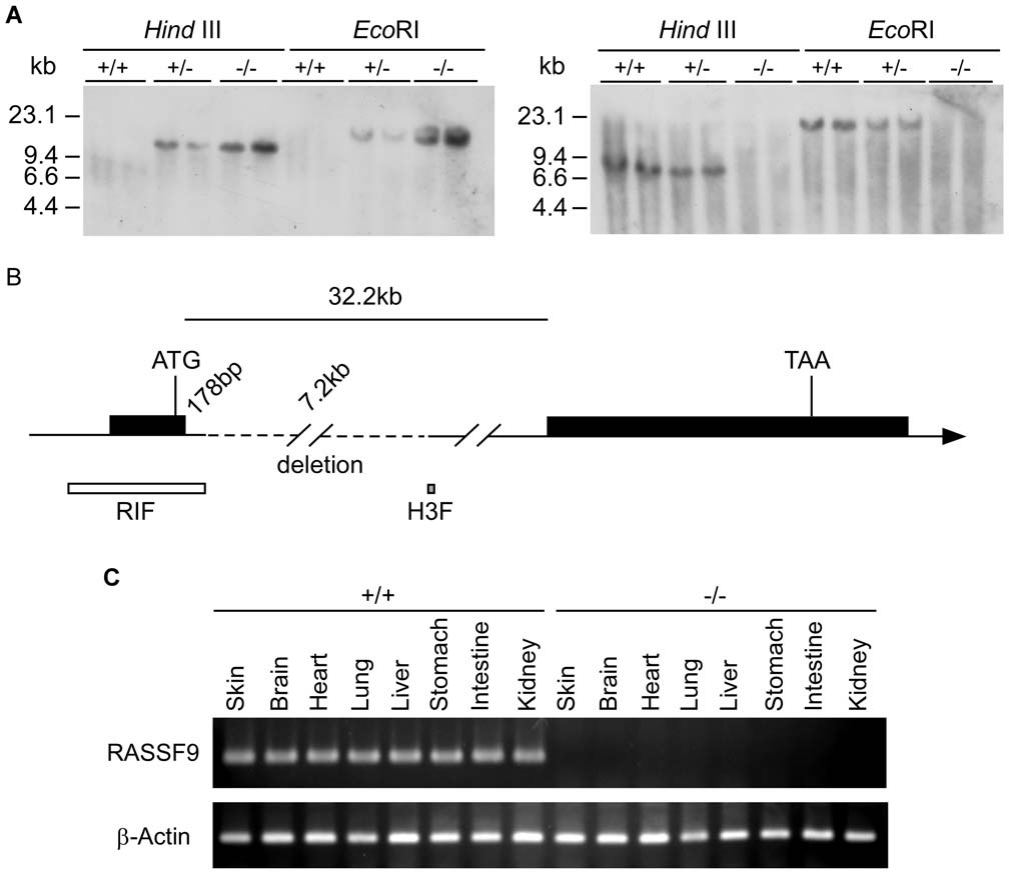
Chromosomal disruption and *RASSF9* gene silence in transgenic mutant line. (A) Southern blot analysis. Genomic DNA samples from mice of various genotypes were separately digested with *Hind*III or *EcoR*I. The left panel showed that a single hybridization band was detected when the transgene was used as the probe. The right panel confirmed a deletion of the identified region in the homozygous mice, as determined by the lack of signal from a probe locating this region. (B) Physical map of the *RASSF9* gene. The sequences flanking the inserted transgene in mutant mice were identified by positional cloning. Symbols: white box, RIF; gray box, H3F; black line, intronic DNA; dashed line, the region deleted by the insertional mutagenesis. (C) Total RNA extracted from the indicated tissues of mice of the WT and *RASSF9*−/− (−/−) genotypes (n = 3 per genotype) were analyzed by RT-PCR using *RASSF9* and β-Actin gene-specific primers. β-Actin was used as the internal control for respective samples. The RT-PCR product was analyzed by 2% agarose gel electrophoresis. Similar results were obtained from two independent experiments in panel A. +/+, wild type; +/−, heterozygotes; −/−; homozygotes.

In order to elucidate the molecular mechanism of the transgene insertion in the mutant mouse line, we determined the chromosomal integration site of transgene by Southern blot analysis using the transgene as the probe (Tg_probe; Supplementary Information, Table S1). A single band was detected upon restriction digestion of the genomic DNA by the enzymes *Hind*III and *EcoR*I, suggesting that the transgene had integrated at a single locus, as the transgene contained no restriction sites targeted by the enzymes (Figure 2A, left panel). In order to identify the chromosomal location of the transgene-insertion site, we used an inverse PCR approach [15]. Two flanking fragments (designated H3F and RIF; Figure 2B) containing short unique DNA sequences that were not part of the transgene were recovered. The flanking DNA fragments, which were assigned to the mouse chromosome 10D1 region, were separated by about 7.2 kb, indicating that integration of the transgene resulted in the deletion of about 7.2 kb of the genome at the insertion site (Figure 2B). To confirm these results, we used the same blot to perform Southern hybridization with a probe located within the putative 7.2-kb deletion (Δ_probe; Table S1); no signal was detected in homozygous mutant (−/−) mice (Figure 2A, right panel). The deleted genomic sequence was mapped to an intron sequence approximately 178 bp downstream of the first exon of the gene encoding *RASSF9* (Figure 2B). The mapping of the deletion/transgenic insertion to murine *RASSF9* gene (accession number: AK041688) was confirmed with the genomic sequence from the latest assembly of the UCSC Genome Browser [July 2007 assembly (NCBI37/mm9); http://genome.ucsc.edu]. Thus, we concluded that the transgenic mice harbored a 7.2-kb chromosomal deletion within the 32.2-kb intron of the RASSF9 gene, in close proximity to its first exon. In this study, we routinely performed genotype screening by genomic PCR using primers targeting the *LMP*-1 transgene and the deleted sequence of the *RASSF*9 intron (Figure S2A, bottom two panels). Further analyses of tissue samples from homozygotes (−/−) demonstrated that the chromosomal deletion resulted in *RASSF9* gene silencing in multiple organs, as confirmed by reverse-transcription PCR (RT-PCR; Figure 2C; Figure S2A, the top second panel).

### Perturbation of skin architecture in *RASSF9*−/− mice

**Figure 3:**
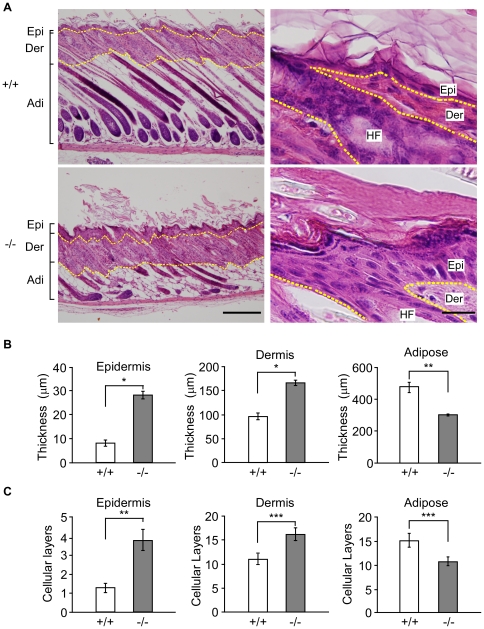
Histological abnormalities in *RASSF9*−/− skin. (A) H&E staining of skin sections from two-week-old mice. The dashed yellow lines denote the epidermis-dermis and dermis-adipose borders. Similar results were obtained from three independent pairs of mice. The low and high magnification images were shown in the left and right panels, respectively. Left panel, scale bar  = 200 µm; Right panel, scale bar  = 20 µm. Epi, epidermis; Der, dermis; Adi, adipose; HF, hair follicle. (B, C) Quantitative analysis of the thickness and cellular layers of the epidermis, dermis and adipose tissues of histological skin sections from ten individual regions of each genotyping mouse at two weeks after birth. Values were measured by thickness and numbers of cellular layer in panel (B) and (C), respectively. Mean ± SD (n = 3, per genotype); * *P*<0.001, ** *P*<0.005, *** *P*<0.05. Panel A–C: +/+, wild type; +/−, heterozygotes; −/−, homozygotes.

Since homozygous mutant mice showed phenotypes typical of the alopecia syndromes, we next sought to characterize its skin pathology. Hematoxylin and eosin (H&E) staining of fixed skin tissue sections revealed thickening of the epidermis and dermis of *RASSF9*−/− skin by ∼3-fold and 1.7-fold, respectively, versus that of the WT mice at two weeks old (Figure 3A and B). The histological features of dermal and epidermal thickening of *RASSF9*−/− skin could also be observed with Masson's trichrome staining, which stained collagen blue for better contrast of collagen-rich dermis with collagen-free epidermis (Figure S3). On the other hand, the subcutaneous adipose tissue of the mutant mouse was only 60% of that of the WT mouse (Figure 3A and B; Figure S3). In the mouse, the hair cycle consists three stages: anagen (hair growth), catagen (apoptosis-driven regression), and telogen (resting maintenance). Strikingly, in contrast to anagenic WT hair follicles, the majority of the mutant hair follicles were at the catagen and telogen phases, with many of them devoid of hair shafts [Figure 3A and Figure S3, lower left panels; contrast with upper left (WT) panels]. These observations strongly suggest that *RASSF9,* the gene disrupted by the transgene insertion in this mutant line, is crucial for epidermal development.

### Aberrant proliferation in skin of *RASSF9*−/− mice

**Figure 4:**
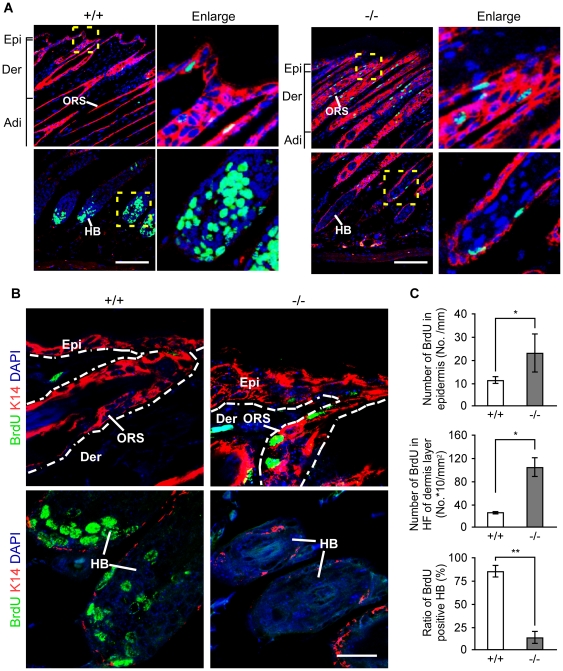
Aberrant proliferation in *RASSF9*−/− skin. (A) BrdU incorporation (green) and K14 (red) were co-stained by immunofluorescence in the skin cross-sections of two-week-old *RASSF9*−/− (−/−; right panel) and WT mice (+/+; left panel). Areas marked by yellow dashed line are enlarged in the adjacent column (“Enlarge”). Scale bar  = 100 µm. ORS, outer root sheath; HB, hair bulb. The abbreviations Epi, Derm, Adi were defined as described in Figure 3A. (B) High-magnification images of BrdU incorporation in dermal tissues. Abnormal patterns of proliferating cells in Epi, ORS and HB of −/− skin are consistent with those observed in (A). The dashed white lines denote the epidermis-dermis border. Similar results were obtained from three independent pairs of mice. Scale bar  = 20 µm. (C) Skin proliferation was evaluated by quantitation of BrdU incorporation using ImageJ software. Analyses were performed for the number of BrdU-positive nuclei in basal layer (top panel; per mm of epidermis); the number of BrdU-positive nuclei in dermal hair follicles (middle panel; per mm^2^ of dermis layer); and the percentage of HB with more than five BrdU-positive nuclei (bottom panel). Data represent scoring of at least 20 hair bulbs from each mouse and three mice for each genotype. *, *P*<0.05; **, *P*<0.001.

The skin of *RASSF9*−/− mouse exhibited a significant epidermal thickening. We therefore investigated whether RAFF9 affected the epidermal proliferation by determining bromodeoxyuridine (BrdU) incorporation in the skin of two-week-old mice. BrdU, the thymidine analog, incorporates into replicating DNA; it is useful as a label of proliferating cells. Indeed, immunofluorescence staining for BrdU-incorporated cells in skin tissues revealed marked changes of cellular proliferation patterns in the basal layers and hair follicles of *RASSF9*−/− mice versus WT. (Figure 4A and B). BrdU incorporation doubled in the epidermal basal layer of the *RASSF9*−/− mice versus WT control (Figure 4A and B; 4C, top panel). Additionally, BrdU-positive cells were 4-fold more in the outer root sheath (ORS) of the hair follicles located at the region of dermis layer in *RASSF9*−/− mice (Figure 4A and B; 4C, middle panel). However, the BrdU-labeling hair bulb index, defined by the ratio of the number of hair bulb (HB) with more than five BrdU-immunolabeled nuclei to total number of HB, was reduced to ∼15% of the WT control (Figure 4A and B; 4C, bottom panel), revealing a remarkable loss of anagenic follicles in *RASSF9*−/− mice. Taken together, these findings suggest adverse effect on epidermal homeostasis and hair-cycle regulation caused by disruption of the *RASSF9* gene.

**Figure 5:**
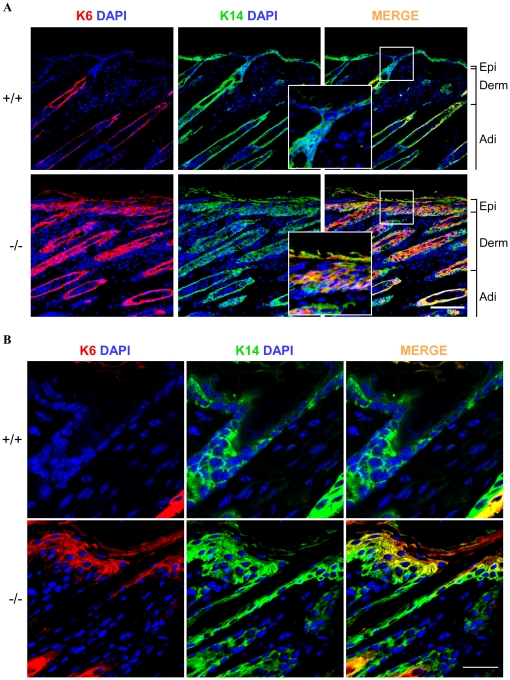
K6 is abnormally expressed in epidermis of *RASSF9*−/− skin. (A) Frozen sections of skin from two-week-old mice were double-immunofluorescence stained for K6 (red) and K14 (green), with the merged signals in yellow. DAPI (blue) was used to for nuclear staining. Scale bar  = 100 µm. Similar results were obtained from three independent mice. +/+, wild type; −/−, *RASSF9*−/−. The other abbreviations of Epi, Derm, Adi were defined as described in Figure 3A. Areas outlined in white borders were enlarged for detailed views (insets). (B) High-magnification images of K6 and K14 expression, prepared as described above. Scale bar  = 20 µm.

We also examined the expression of keratin 6 (K6), a marker of hyperproliferative epidermis, whose expression is highly restricted to a single cell layer in the hair follicle that surrounds the innermost layer of the outer root sheath, but otherwise not typically expressed in epidermis [16], [17], [18]. In *RASSF9*−/− mice, K6 was highly expressed throughout the epidermal layers and entire root sheath of hair follicles in abnormally thick layers of cells (Figure 5). Keratin 14 (K14), a marker of epidermal proliferating compartment, was confined to the basal cells in WT skin as expected, whereas it was also detected in the suprabasal layer of *RASSF9*−/− mice, also abnormally thick, together with K6 (Figure 5). The striking co-expression of K6 and K14 in abnormally thickened epidermal tissues suggests hyperproliferation in the epidermis of two-week-old *RASSF9*−/− mice (Figure 5).

### Aberrant differentiation in skin of *RASSF9*−/− mice

**Figure 6:**
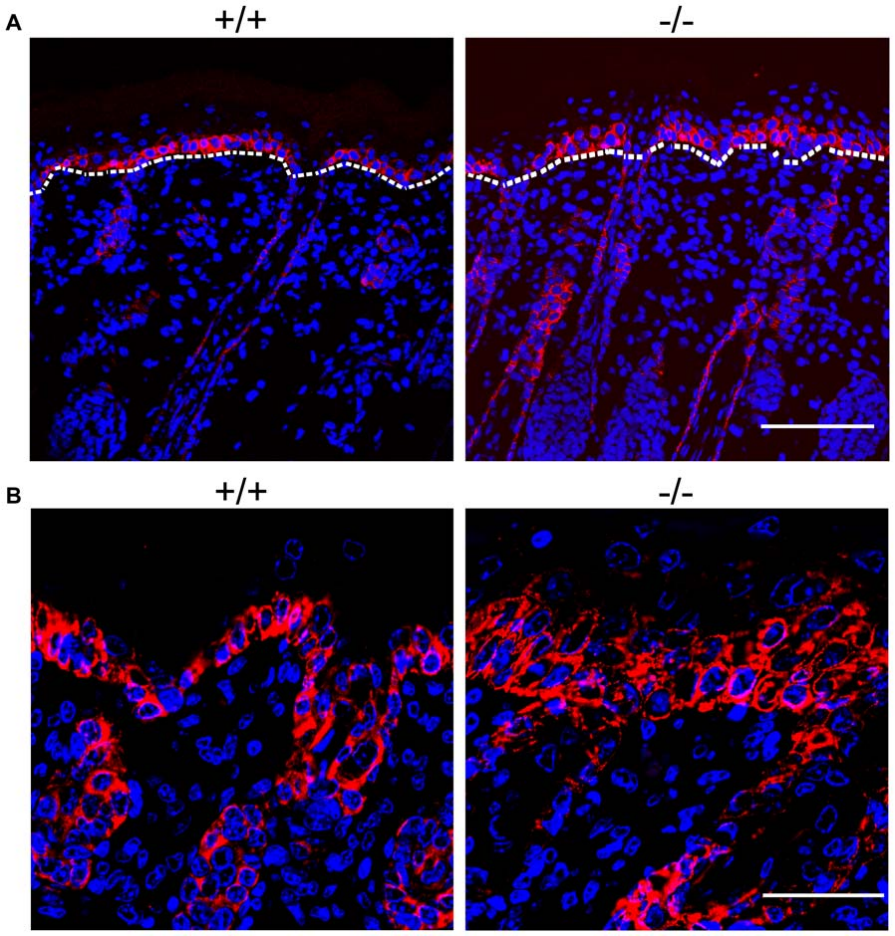
Aberrant differentiation in the skin of *RASSF9*−/− mice—K5 (red). (A) Frozen sections of dorsal skins of WT (+/+) and *RASSF9*−/− (−/−) mice were immunostained with red fluorescence for K5 in 4-dpp mice. Scale bar  = 100 µm. (B) Images at higher magnification. Scale bar  = 40 µm. Blue, DAPI staining of cell nuclei.

**Figure 7:**
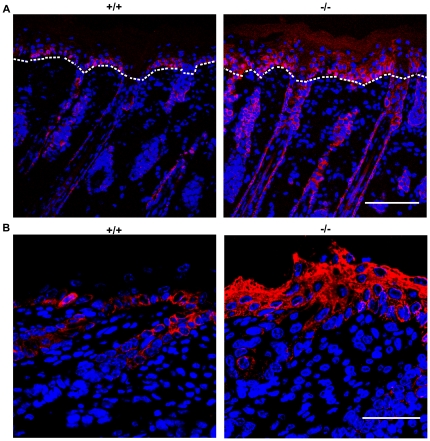
Aberrant differentiation in the skin of *RASSF9*−/− mice—K14 (red). (A) Frozen sections of dorsal skins of WT (+/+) and *RASSF9*−/− (−/−) mice were immunostained with red fluorescence for K14 in 4-dpp mice. The dashed white lines denote the epidermis-dermis border. Similar results were obtained from three independent pairs of mice. Scale bar  = 100 µm. (B) Images at higher magnification. Scale bar  = 40 µm. Blue, DAPI staining of cell nuclei.

**Figure 8:**
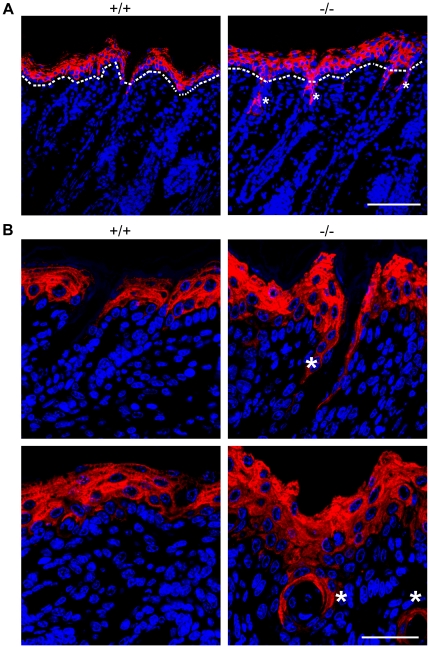
Aberrant differentiation in the skin of *RASSF9*−/− mice—K1 (red). (A) Frozen sections of dorsal skins of WT (+/+) and *RASSF9*−/− (−/−) mice were immunostained with red fluorescence for K1 in 4-dpp mice. The dashed white lines denote the epidermis-dermis border. Similar results were obtained from three independent pairs of mice. Scale bar  = 100 µm. Note the abnormal staining of K1 expression of hair follicular cells (*) in dermis of *RASSF9*−/− skin. (B) Images at higher magnification, with follicular sites of abnormal K1 expression identified by asterisks (*) in both length-wise sections (top panels) and cross sections (bottom panels) of hair follicles. Scale bar  = 40 µm. Blue, DAPI staining of cell nuclei.

**Figure 9:**
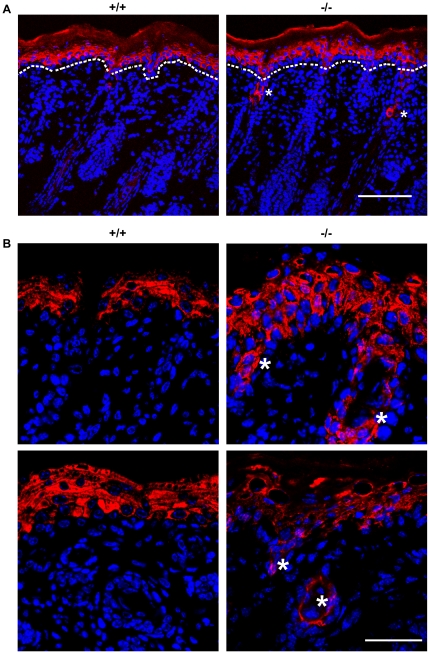
Aberrant differentiation in the skin of *RASSF9*−/− mice—K10 (red). (A) Frozen sections of dorsal skins of WT (+/+) and *RASSF9*−/− (−/−) mice were immunostained with red fluorescence for K10 in 4-dpp mice. The dashed white lines denote the epidermis-dermis border. Similar results were obtained from three independent pairs of mice. Scale bar  = 100 µm. Note the abnormal staining of K10 expression of hair follicular epithelium (*) in dermis of *RASSF9*−/− skin. (B) Images at higher magnification, with follicular sites of abnormal K10 expression identified by asterisks (*) in both length-wise sections (top panels) and cross sections (bottom panels) of hair follicles. Both K1 and K10 are markers of early-stage keratinocyte differentiation, suggesting a delay in epidermal and follicular keratinocyte maturation. Scale bar  = 40 µm. Blue, DAPI staining of cell nuclei.

**Figure 10:**
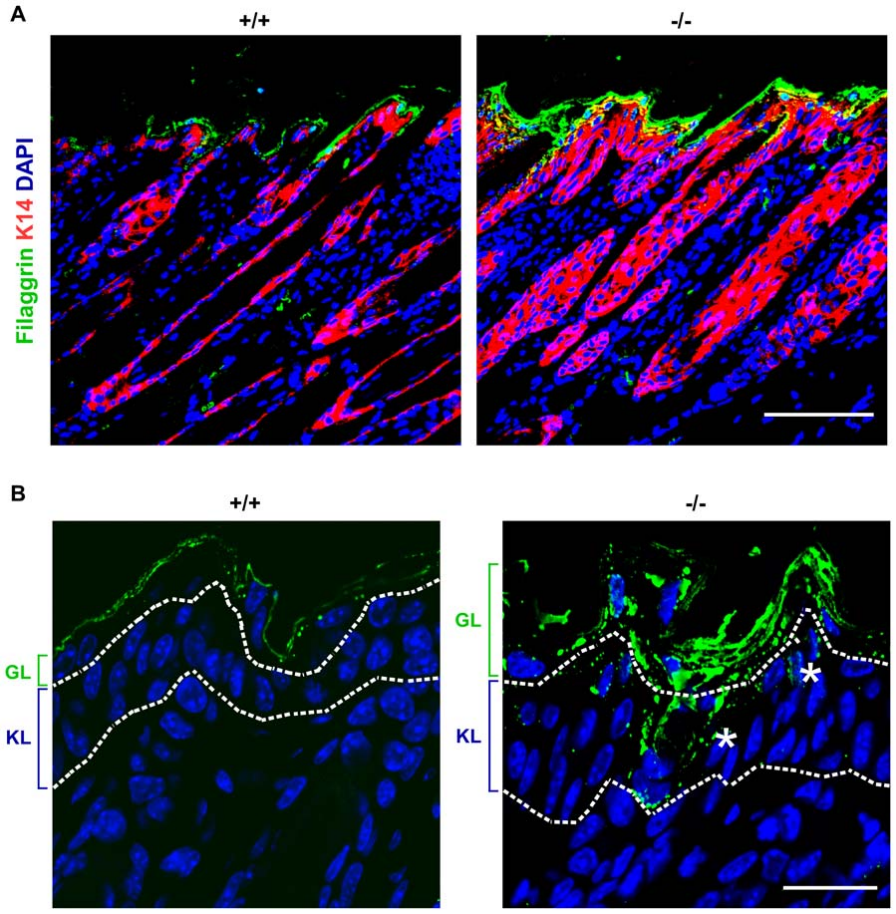
Aberrant differentiation in the skin of *RASSF9*−/− mice—filaggrin (green). (A) Frozen sections of dorsal skins of *RASSF9*−/− and WT control mice were immunostained with green fluorescence for filaggrin and red fluorescence for K14 in two-week-old mice. Blue, DAPI staining of cell nuclei. Scale bar  = 100 µm. (B) Images of higher magnification for filaggrin staining (green fluorescence) in skin sections. The dashed white lines denote the epidermis-dermis border. Similar results were obtained from three independent pairs of mice. Scale bar  = 20 µm; GL, granular layer; KL, keratinocyte layer. Note the increased thickness and patchiness in layer of prominent filaggrin staining, as well as abnormal distribution of filaggrin staining in keratinocyte layer and hair follicle (*).

Epidermal homeostasis of the skin requires a coordinated regulation of cell proliferation and differentiation. Thus we investigated the status of epidermal differentiation in *RASSF9*−/− mice. We performed immunofluorescence staining for keratin 5/keratin 14 and keratin 1/keratin 10 (K5/K14 and K1/K10), markers for the basal and suprabasal layers of the cutaneous skin, respectively [19]. In *RASSF9*−/− mice at 4 days post-partum (dpp), K5 and K14 were detected not only in the basal layer but also the suprabasal layers of epidermis, abnormal patterns that echoed the epidermal hyperplasia observed of two-week-old *RASSF9*−/− mice (K5: Figure 6; K14: Figure 7). Moreover, detection of K1/K10 expression revealed drastic thickening of suprabasal layers in *RASSF9*−/− mice, in contrast to the WT control (K1: Figure 8; K10: Figure 9). Furthermore, aberrant expression of K1 and K10 was observed in *RASSF9*−/− follicles, an anomaly strongly suggesting possible epidermalization of the hair follicles in the skin of *RASSF9*−/− mice (Figures 8–9; detailed images of hair follicles in 8B and 9B). Finally, immunostaining of filaggrin, a marker expressed by cells of granular layer, revealed abnormal expression of filaggrin in multiple layers of granular cells beneath the stratum corneum of *RASSF9*−/− mice at two weeks of age, in contrast to the thin-layer pattern typically maintained in the granular layer of WT skin (Figure 10; detailed images in 10B). Taken together, these findings showed that *RASSF9*−/− mice suffered from a severe defect in epidermal homeostasis characterized by abnormal thickening of epidermis, dysregulated cellular proliferation, and disruption of keratinocyte maturation as revealed by the altered patterns of keratin and filaggrin expressions.

### RASSF9 expression profiles in WT mouse

**Figure 11:**
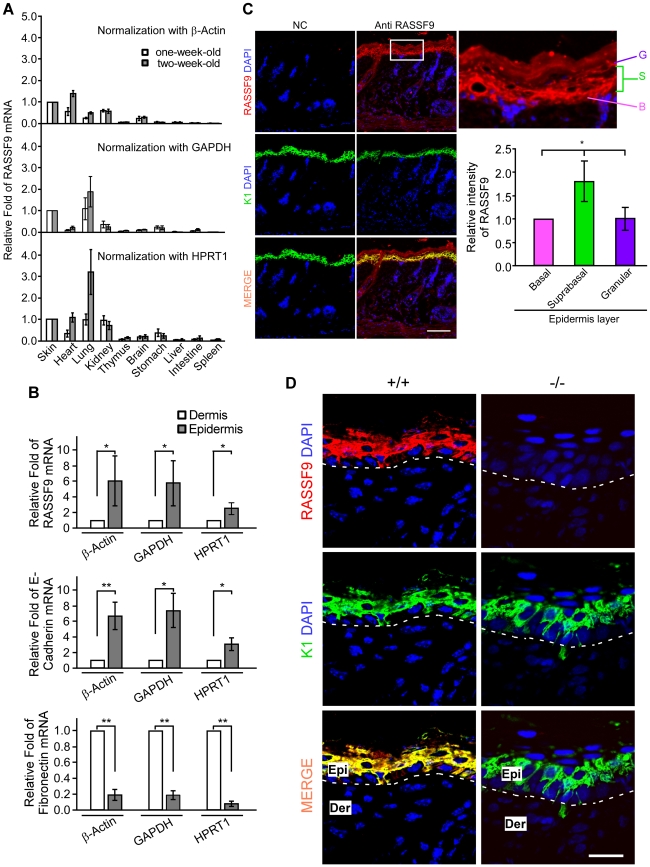
RASSF9 expression profiles in WT mouse. (A) Quantitive RT-PCR analysis of *RASSF9* mRNA expression in various tissues of WT mice at one and two weeks old. The results were normalized with regard to the mRNA expression of β-Actin (top panel), *GAPDH* (middle panel), or *HPRT1* (bottom panel). Values represent the mean ± SD (one-week-old mice, n = 4; two-week-old mice, n = 5). (B) Quantitive RT-PCR analysis of RASSF9 (top panel), E-cadherin (*CDH1*) (middle panel), and fibronectin (*fn1*) (bottom panel) mRNA expression in the epidermis and dermis of the WT skin at 4-dpp old. The results were normalized to individual reference gene of β-Actin, *GAPDH*, or *HPRT1* (the reference gene is specified at the bottom). The results were normalized with regard to the mRNA expression of β-Actin, *GAPDH*, or *HPRT1*. Values represent the mean ± SD (n = 4). *P<0.05, **P<0.001. (C) Frozen sections of 4-dpp skins were double-stained for RASSF9 (Red) and K1 (Green), with merged signals in yellow. “NC”, negative control, with no incubation in anti-RASSF9 anti-serum; immunostaining procedures for NC were otherwise identical to “anti-RASSF9” panels. Blue, DAPI staining of cell nuclei. Scale bar  = 100 µm. Area in white frame was enlarged to show the RASSF9 location in the WT skin (upper right panel). The relative intensity of RASSF9 in various epidermal layers of WT skin at 4-dpp were normalized to basal-layer signals (lower right panel). Data represent analyses by ImageJ of five individual images from each mouse (n = 3) and are presented as Mean ±SD fold intensity relative to basal layer. *, *P*<0.05. B, basal layer; S, suprabasal layer; G, granular layer. (D) Higher-magnification images of K1 (green) and RASSF9 (red) co-staining in frozen sections of dorsal skins of WT (+/+) and *RASSF9*−/− (−/−) mice. No RASSF9 signal was detected in *RASSF9*−/− epidermis. The dashed white lines denote the epidermis-dermis border. Similar results were obtained from three independent pairs of mice. Scale bar  = 20 µm. Epi, epidermis; Der, dermis.

To further evaluate the significance of *RASSF9* gene expression for normal mouse development, we determined the expression profiles of *RASSF9* gene in various organs of WT mice at one or two weeks old. Our results indicated that the *RASSF9* mRNA was expressed in multiple organs, with high-level expression seen in the skin, moderate-to-high expression in the heart, lung and kidney, and relatively low expression in the thymus, brain, stomach, liver, intestine and spleen (Figure 11A). Interestingly, RASSF9 mRNA expression in heart and lung increased with growth from one to two weeks old, implicating a role of this gene in regulation of post-partum maturation of these organs in addition to epidermis development. We then isolated epidermal and dermal fractions from the skin of 4-dpp WT mice and examined the site-specific expression of *RASSF9* mRNA in skin. The *RASSF9* mRNA was between two- and six-fold higher in the epidermal fraction than the dermal fraction, depending on the reference gene used (Figure 11B, top panel). The demarcation of epidermis and dermis layers was confirmed by specific detection of mRNA for E-cadherin (*CDH1*) and fibronectin (*fn1*) in the former and latter fractions, respectively (Figure 11B, middle and bottom panels).

We further visualized the expression patterns of RASSF9 protein in mouse skin tissues by double immunofluorescence staining of frozen sections using RASSF9- and K1-specific antibodies. Western immunoblot detection of exogenously expressed RASSF9 confirmed the specificity of anti-RASSF9 antiserum (Figure S1). The RASSF9-specific signal was detected throughout the entire epidermis of wild type (WT, +/+) mice at 4-dpp while colocalizing with K1 in the suprabasal layer (Figure 11C; higher-magnification images in Figure 11D). Immunofluorescence signals specific for RASSF9 were not detected in the *RASSF9*−/− skin section, again demonstrating the specificity of the anti-RASSF9 antiserum [Figure 11D; compare +/+ (left panels) with −/− (right panels)]. Quantification of the RASSF9 immunofluorescent intensity using ImageJ software revealed a more prominent expression of RASSF9 in suprabasal layers, compared to that in the basal and granular layers (Figure 11C). *In situ* hybridization with an antisense probe against the *RASSF9* mRNA further confirmed strong and specific expression of *RASSF9* that is prominent in suprabasal layer of normal mouse epidermis (Figure S4).

### RASSF9 affects keratinocyte growth and differentiation *in vitro*

**Figure 12:**
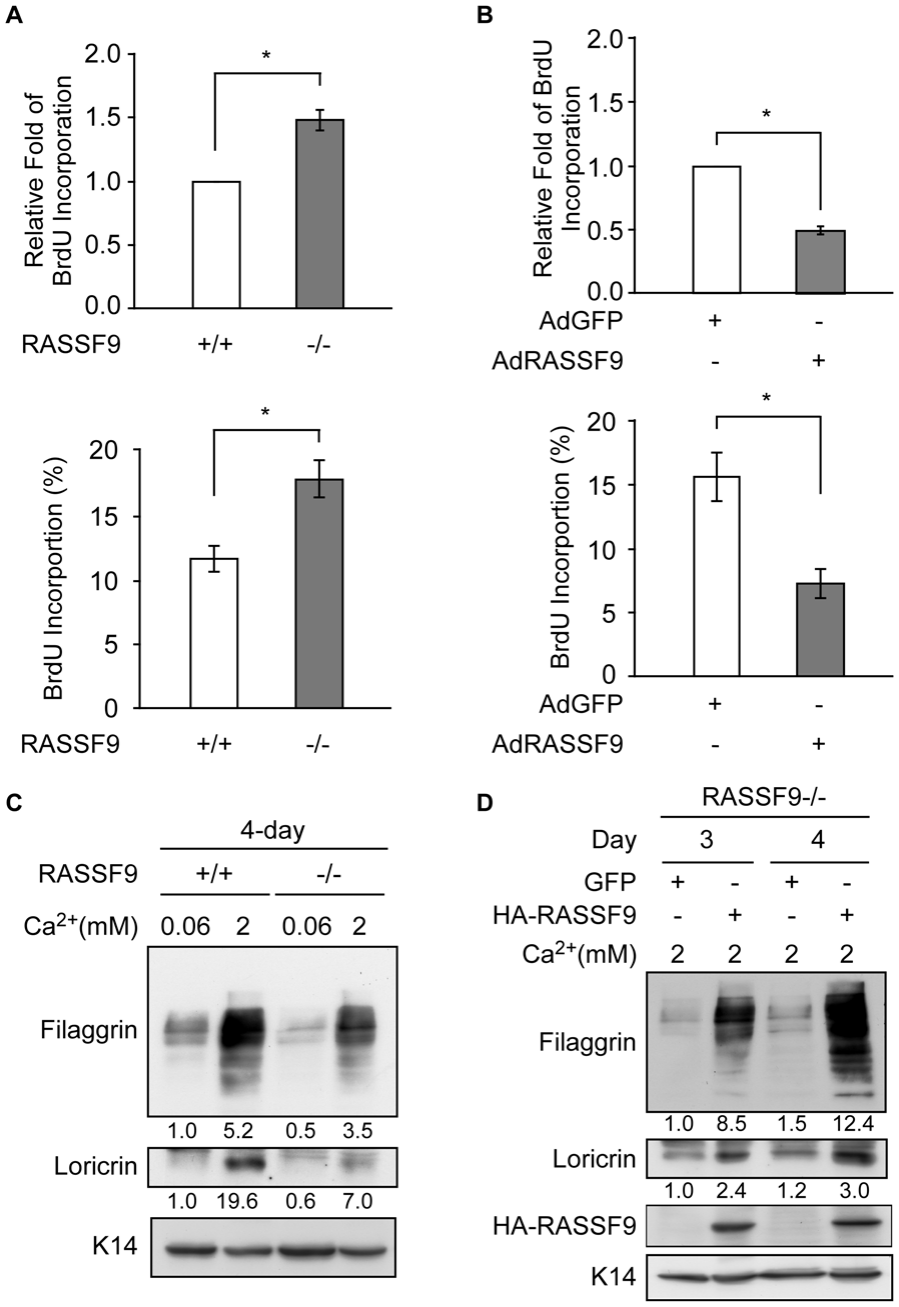
RASSF9 affects keratinocyte proliferation and differentiation. (A, B) RASSF9-mediated proliferation measured by BrdU incorporation using a Cell Proliferation Biotrak ELISA kit (Amersham) and BrdU immunofluorescence. The BrdU incorporation rate was normalized to the amount of input protein or nuclei. Values are shown as fold changes versus the control group or percentage of nuclei presented in top panel and bottom panel, respectively. (A) BrdU incoporation rate was performed in mouse primary keratinocytes after two-day incubation under growth medium. Mean ± SD (n = 3). *, *P*<0.05. (B) BrdU incoporation rate was evaluated from *RASSF9*−/− keratinocytes expressing HA-RASSF9 versus GFP control by adenoviral post transduction on 1 day and then subjected to growth medium for 2 days. Mean ± SD (n = 2). *, *P*<0.05. (C, D) The terminal differentiation of keratinocytes was analyzed by immunoblotting with specific antibodies against filaggrin and loricrin. K14 was used as loading control. Total cell extracts were prepared from mouse primary keratinocytes incubated for the indicated time under condition for growth (0.06 mM calcium) or differentiation (2 mM calcium). The intensity of protein expression was determined as the density of the relevant band normalized to that of the K14 loading control, as determined by ImageQuant 5.1. The resultant values were further normalized to baseline (lane 1 of same blotting) and shown below the images. Similar results were obtained from three independent experiments. (C) Primary *RASSF9*−/− and WT mouse keratinocytes incubated in the indicated medium for 4 days. (D) Primary *RASSF9*−/− keratinocytes transduced with Adv/HA-*RASSF9* or a GFP-expressing control virus and then subjected to differentiation induction for 3 and 4 days. Panel B and D: +/+, wild type; −/−, *RASSF9*−/−.

Since RASSF9 is expressed in the proliferating basal and differentiating suprabasal epidermal layers, it may have significant involvement in keratinocyte proliferation and differentiation. Therefore, we isolated primary keratinocytes from *RASSF9*−/− mice and measured their BrdU incorporation. A moderate (1.5-fold) but statistically significant increase in BrdU incorporation (*p*<0.05) was detected in *RASSF9*−/− keratinocytes versus WT keratinocytes, in terms of both extent of BrdU incorporation and the percentage of BrdU-positive cells (Figure 12A). Conversely, re-expression of RASSF9 by infection with the Adv/HA-*RASSF9* recombinant virus inhibited the BrdU incorporation in *RASSF9*−/− keratinocytes (*P*<0.05; Figure 12B). These results show that RASSF9 is involved in suppressing or regulating proliferation of epidermal keratinocytes. As described above, RASSF9 expression was prominent in suprabasal layers of epidermis, and *RASSF9* null mice exhibited aberrant differentiation. These observations prompted us to test whether RASSF9 was actively involved in keratinocyte differentiation. A high concentration of calcium (2 mM) was used to induce the differentiation of primary mouse keratinocytes in culture [10], and two markers, loricrin and filaggrin, were used for evaluation of terminal differentiation. We found that both loricrin and filaggrin were expressed at lower levels in *RASSF9*−/− versus WT keratinocytes, regardless of calcium concentrations and incubation lengths tested (4 days, Figure 12C; 2 days, Figure S5A). In cells cultured in 2 mM calcium for 4 days, the levels of filaggrin and loricrin in *RASSF9*−/− keratinocytes were about two-third and one-third, respectively, of those in WT keratinocytes (Figure 12C). These results suggest that the terminal differentiation process of *RASSF9*−/− keratinocytes is deficient.

To confirm that the defects in the induction of terminal differentiation in *RASSF9*−/− keratinocytes were due to the loss of RASSF9, we rescued RASSF9 expression by transducing cells with Adv/HA-*RASSF9*, and examined the differentiation of transduced cells. Our results revealed that filaggrin and loricrin were induced by approximately 8- and 2.5-fold in cells that re-expressed RASSF9 versus those expressing GFP (control), when the cells were cultured under the differentiation-inducing conditions (2 mM calcium) for 3 or 4 days (Figure 12D). Notably, expression induction of filaggrin and loricrin by ectopic RASSF9 expression in *RASSF9*−/− keratinocytes was also observed in growing condition (0.06 mM calcium) for 2 days (Figure S5B), suggesting that RASSF9 expression intrinsically primed cells for initial differentiation independent of calcium levels. Taken together, these findings suggest that RASSF9 is required for proper initiation of keratinocytes differentiation.

### RASSF9 modulates p21Cip1 induction in keratinocytes

**Figure 13:**
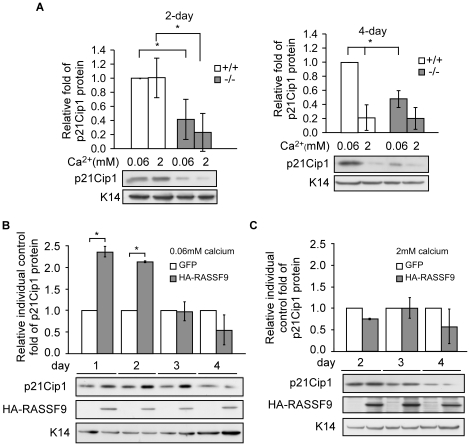
The effect of RASSF9 on p21Cip1 expression in keratinocytes. (A) *RASSF9*−/− and WT mouse keratinocytes were cultured in growth (0.06 mM calcium) and differentiation-inducing (2 mM calcium) medium for 2 days (left panel) and 4 days (right panel) prior to lysis and immunoblotting with the indicated antibody. K14-normalized intensity of p21Cip1 protein signal was determined by ImageJ software. The results were further normalized to baseline control (lane 1 of same blotting) and shown on the top of blot images. Mean ± SD (n = 3). *, *P*<0.05; +/+, wild type; −/−, *RASSF9*−/−. (B) Expression by transduction of HA-RASSF9 or GFP as negative control in *RASSF9*−/− cells under the growth condition (0.06 mM calcium) was examined for indicated time points. Immunoblotting and data analysis were performed as described in (A). Fold intensities relative to respective GFP controls are shown on the top of blot images. Mean ± SD (n = 2). *, *P*<0.05. (C) Re-expression of RASSF9 in *RASSF9*−/− cells under differentiation-inducing condition. Recombinant adenovirus infection, cell-lysate harvesting, immunoblotting and data analysis were performed as described in (B), except cells were subsequently incubated in 2 mM calcium medium for 2, 3 and 4 days on post transduction. Mean ± SD (n = 3).

To gain insight into the potential involvement of cell-cycle regulation in the RASSF9-mediated growth of keratinocytes, we compared the Affymetrix microarray analysis data of gene expression patterns between *RASSF9*−/− and WT keratinocytes cultured in the growth medium. P21Cip1 (*CDKN1A*) was identified among several transcripts shown to be down-regulated in *RASSF9*−/− cells. The protein is a potent inhibitor of cyclin-dependent kinases and is known to be a key checkpoint protein for the transition of keratinocyte growth/differentiation [20]. To test whether the expression levels of p21Cip1 varied in keratinocytes cultured under growth (0.06 mM calcium) or differentiation-inducing (2 mM calcium) conditions, we examined p21Cip1 expression by Western blot analysis. Although the p21Cip1 level was significantly lower in *RASSF9*−/− keratinocytes compared to WT keratinocytes when cells were cultured in the growth medium (0.06 mM calcium) for 2 or 4 days (approximately 60% and 50% of WT, respectively; Figure 13A), this difference gradually decreased when cells were cultured in the differentiation-inducing medium (2 mM calcium) from day 2 to day 4 (from 75% to 0%; Figure 13A). After 4 days of culture in the high-calcium medium, the cells further progressed toward the terminal differentiation stage; this was accompanied by a dramatic reduction of p21Cip1 protein levels in both *RASSF9*−/− and WT keratinocytes (Figure 13A, right panel). High calcium induces terminal differentiation of keratinocytes that is coincident with a more dramatic reduction of p21Cip1 [21], which can be used as an indicator of progression of keratinocytes toward terminal differentiation in this study. Thus, these results suggest that RASSF9 affects p21Cip1 expression primarily in keratinocytes that are at the stages of growth or early differentiation. To further test whether RASSF9 can modulate p21Cip1 expression in keratinocytes as they progress from growth to differentiation, we maintained in low-calcium condition *RASSF9*−/− keratinocytes transduced to express recombinant RASSF9. RASSF9 expression significantly induced p21Cip1 expression by 2.3-fold on day 1, but the fold-change diminished thereafter over time (Figure 13B). However, p21Cip1 induction in RASSF9-re-expressing *RASSF9*−/− cells did not occur under differentiation-inducing condition (Figure 13C). These results further support the hypothesis that RASSF9 affects p21Cip1 expression primarily in growth-phase or early-differentiating keratinocytes. Taken together, these findings strongly suggest that the reduction of p21Cip1 in *RASSF9*−/− keratinocytes potentially form the basis for the cellular hyperproliferation observed in the epidermis of *RASSF9*−/− mice.

## Discussion

The N-terminal (NT) RASSF protein family is an evolutionarily conserved group that has orthologs in the lower vertebrate, *Xenopus*, and the invertebrate, *Drosophila* [1], [4]; these genes are believed to potentially function in development. We first found that RASSF9 was predominately expressed in the stratified epithelium, where its function appeared to be linked to epidermal homeostasis. Indeed, absence of RASSF9 expression resulted in atypical histological architecture of the skin of *RASSF9*−/− mice, characterized by changes in thickness of histological architecture and abnormal development of hair follicles. In addition, aberrant distributions of various keratin markers for different stages of keratinocyte differentiation was observed in *RASSF9*−/− skin, indicating an inability to maintain a regulated program of epidermis development.

The stratum corneum can provide a mechanical barrier to protect the body from the environmental damage and dehydration; maintenance of epidermal development and homeostasis is thus critical for its proper function. The various anomalies observed in the skin of neonatal *RASSF9*−/− mice may therefore culminate in defective skin barrier function. In fact, assessment of skin permeability using the toluidine blue penetration assays showed that approximately 17% of heterozygous pups and 40% of homozygous pups exhibited moderately increased dye permeability of skin with a punctuated distribution of blue spots in *RASSF9*−/− mice versus WT control suggesting slightly compromised skin barrier for the *RASSF9*-deficient mice (Figure S6). In vitro study of the role of RASSF9 in epidermal keratinocyte proliferation and differentiation found that *RASSF9*−/− keratinocytes exhibited diminished or delayed response to differentiation induction by high calcium concentration, with attendant preference for hyperproliferation. Furthermore, adenovirus-mediated re-expression of RASSF9 could overcome those defects of the observed block in the terminal differentiation or hyperproliferation of *RASSF9*−/− cells. Based on these findings, we conclude that RASSF9 is critical for the regulation of skin epithelial proliferation and differentiation. To our knowledge, this is the first direct observation of the novel role of RASSF9 in maintaining homeostasis of cellular proliferation and differentiation.

Although the RASSF9-knockout mice strain was generated by a transgenic insertion of the open-reading frame of an exogenous recombinant gene—EBV LMP-1—we believe that gene silencing by an intron disruption is not implausible. Various intronic regulatory elements have been found to be critical for regulation of gene expression [22], [23], [24], [25], [26]. In addition, the 7.2-kb intron sequence replaced by the transgenic insertion contains a 150-bps long sequence of purine-rich guanine-adenosine (GA) repeats possibly required for proper splicing of the primary transcript [27]. Indeed, we have consistently detected no presence of RASSF9 mRNA or protein expression in *RASSF9*−/− tissues. At the same time, neither mRNA transcripts of the LMP-1 transgene or its protein product was present at detectable level for the length of time frame in which the experiments were performed. Moreover, overexpression of LMP-1 alone by in vitro transduction of primary keratinocytes at a level drastically greater than the observed level in tissue samples resulted in no altered filaggrin expression levels (Figure S7). Even as it is likely that abrogated RASSF9 expression directly contributed to the observed phenotypes, the link between RASSF9 and the observed phenotypes may well be conclusively established by targeted knockout of the RASSF9 allele.

The *RASSF9*−/− mouse line showed rapid death at two weeks old, and the histological analysis revealed a severe defect of pulmonary alveoli occurrence, marked by deficient septation of saccules that results in reduced number of alveolar sacs whose size are however grossly enlarged, as well as significant thickening of alveolar epithelium (Figure S8). Thus, RASSF9 was crucial for the maturation of organogenesis. The defective lung development of the *RASSF9*−/− mice is likely the major causality of their failure to thrive and premature death. Unexpectedly, we also observed a slight decrease in the survival rate of WT mice progeny of a heterozygous mother (+/−) before weaning that may be attributed to cachexia of the heterozygous mother versus healthy WT mother mice (Figure 1C). Consistent with this phenomenon, we observed normal pups born from the WT mother with a normal survival rate (data not shown).

The terminal differentiation marker, filaggrin, which is normally expressed in the epidermal granular layer of WT skin, was detected in multiple layers in *RASSF9*−/− mice skin (Figure 10). However, *in vitro*-cultured *RASSF9*−/− keratinocytes were found to have significant defects or delays in terminal differentiation. This discrepancy could be explained by *RASSF9*−/− cells having an unusual proliferative capacity *in vivo*, which might cause multiple granular layers in epidermis by cell accumulation with a characteristic of delayed differentiation.

RASSF9 is expressed throughout the entire epidermis of WT skin, which consists of the basal layer of growing keratinocytes and the suprabasal layer of differentiating keratinocytes (Figure 11C and D; Figure S4). This further supports our contention that RASSF9 plays a critical role in epidermal homeostasis. We showed that the re-expression of RASSF9 in *RASSF9*−/− keratinocytes could induce p21Cip1 expression and reduce DNA synthesis. Indeed, quantitative analysis of p21Cip1 (*CDKN1A*) mRNA expression in mouse skin tissue by QRT-PCR revealed a general decrease of p21Cip1 mRNA in the skin of *RASSF9*−/− mice (Figure S9). Additionally, we also attempted to determine the protein level of p21Cip1 in skin tissue by Western blot, but p21Cip1 protein was not detectable in skin tissue by this method because of less p21Cip1 protein and high nonspecific mask in there. Previous studies have shown that p21Cip1 inhibits DNA replication by associating with cyclin-CDK and binding to PCNA via two distinct functional domains [28], [29]. Reduced p21Cip1 expression, observed in *RASSF9*−/− keratinocytes and skin, is thus likely to contribute to their hyperproliferation. Consistent with this hypothesis, we found that the endogenous levels of *RASSF9* mRNA were initially induced when keratinocytes were exposed to a calcium concentration of 2 mM (differentiation-inducing conditions) for 1 day, but were thereafter reduced as differentiation progressed (Figure S10). Additional attempts to determine endogenous RASSF9 protein expression in keratinocytes and tissues by immunoblot were inconclusive. Nevertheless, we were able to confirm differential RASSF9 expression in skin tissues by other means of biochemistry and immunohistochemistry as described above. These findings suggest that *RASSF9* gene induction is required for triggering keratinocyte growth arrest that facilitates subsequent differentiation.

Although RASSF9 null expression resulted in diminished filaggrin expression indicative of altered or delayed terminal differentiation in mouse primary keratinocytes, the disruption of a single allele (*RASSF9*+/−) was not sufficient to yield this characteristic (Figure S11). Notably, however, heterozygous (*RASSF9*+/−) mice showed signs of haploinsufficiency, with milder versions of the syndromes seen among the *RASSF9*−/− homozygotes (Figure 1A–C). Although overexpression of RASSFF9 by adenoviral transduction in *RASSF9*−/− keratinocytes efficiently rescued filaggrin expression at low multiplicity of infection (MOI = 5), increasing dosages of RASSF9 beyond 5 MOIs were not able to further enhance the filaggrin expression in *RASSF9*−/− keratinocytes (Figure S12A). Additionally, overexpression of RASSF9 by adenoviral transduction in WT (*RASSF9+/+*) mice keratinocytes also did not result in induction of filaggrin expression under high-calcium condition (Figure S12B). This saturating nature of RASSF9-induced filaggrin expression in mouse primary keratinocytes implies a limit on dependence of filaggrin induction on RASSF9, the intrinsic expression level of which is both necessary and sufficient for normal pattern of filaggrin expression. These findings indicate a possibility that RASSF9 may play an important, decisive role in facilitating the initiation of keratinocyte differentiation, rather than reinforcing it.

While we observed altered proliferations and attendant anomalies in keratinocyte differentiation of *RASSF9*−/− tissues and cells in vitro and in vivo, the question remains on the precise nature of the alteration. It remains possible that RASSF9 deficiency results in altered preference for proliferation without damaging the machinery required for initiation and maintenance of the keratinocyte differentiation program, and the observed changes in selected differentiation markers such as filaggrin and loricrin represent the effect of this altered preference. Regardless, it is apparent that RASSF9 directly or indirectly enforces entry of keratinocyte differentiation, and in its absence cells fail to respond properly to signals such as high calcium concentration, which is recognized as an inducer of keratinocyte differentiation [30], [31], [32], [33]. Indeed, in addition to p21Cip1, our preliminary microarray analysis of the gene expression profile of *RASSF9*−/− keratinocytes hint at possible alteration of pathways known to be involved in epidermis development and keratinocyte differentiations. Further study would be necessary to delineate the exact mechanism by which RASSF9 governs the pathways and factors, including p21Cip1, that are required in concert for proper initiation of keratinocyte differentiation.

Taken together, our main findings with regard to RASSF9 in keratinocyte growth and differentiation can be summarized as follows: 1) *RASSF9* mRNA induction in WT keratinocytes was seen at the early stage of differentiation. 2) Overexpression of RASSF9 in *RASSF9*−/− keratinocytes can overcome the defects of differentiation program. 3) Overexpression of RASSF9 in *RASSF9*−/− keratinocytes inhibited hyperproliferation of the cells, which that linked to p21Cip1 induction under early growth conditions. The expression of p21Cip1 is known to be increased in cell populations adjacent to proliferation compartments (i.e., cells that are poised to differentiate). Furthermore, the protein is believed to be an important, p53-independent regulator of differentiation-associated growth arrest [20], [34], [35], [36].

RASSF9 has been reported to associate with Ras proteins [7]. Ras proteins mediate multiple cellular functions, including cell proliferation, differentiation, survival, and apoptosis [37]. Overexpression of Ras in the suprabasal epidermis or skin equivalent has been reported to disrupt normal stratification, induce cell invasion and proliferation, and trigger the development of skin tumors [38], [39], [40]. Additionally, Ras may be associated with the morphology, spreading, growth, and differentiation of keratinocytes [41], [42], [43], [44]. However, high calcium-induced keratinocyte differentiation has been associated with down-regulation of Ras activity during the early stage of differentiation [45]. As we have observed that RASSF9 is transiently induced when 2 mM calcium is used to induce differentiation among keratinocytes, it would be interesting in the future to examine whether RASSF9 binds to endogenous Ras protein targets in keratinocytes under high-calcium treatment, leading to the attenuation of Ras activity during early differentiation in these cells.

**Figure 14:**
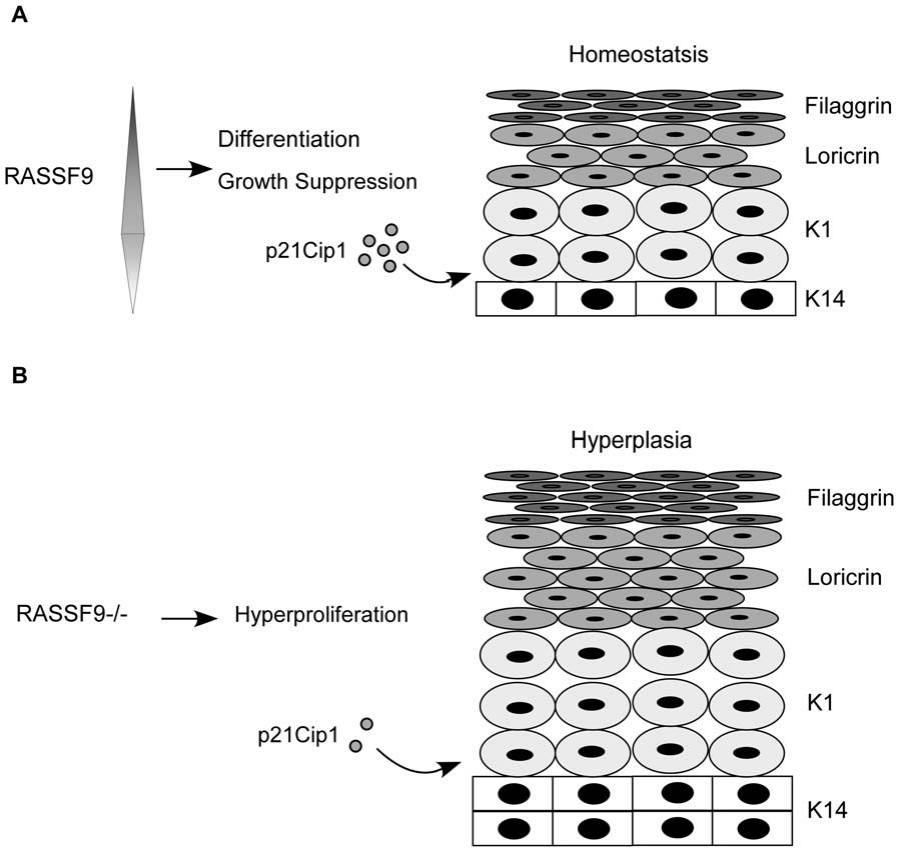
Schematic model of RASSF9-mediated maintenance of epidermal homeostasis through p21Cip1 in keratinocyte growth and differentiation. (A) In WT keratinocytes, RASSF9 expression is induced in cells progressing through early differentiation, but decreases thereafter as cells approach terminal differentiation. RASSF9 induces p21Cip1 expression in keratinocytes during growth and early differentiation, which may mediate in differentiation-related growth arrest. (B) A scheme illustrating that down-regulation of p21Cip1 expression, due to the loss of RASSF9 signaling in post-mitotic cells adjacent to the proliferation compartment, leads to the escape of keratinocytes from cell-cycle withdrawal and their subsequent proliferation.

Based on our present findings, we propose a dynamic model of RASSF9 equilibration in keratinocytes at different differentiation stages (Figure 14). In this model, the profile of the endogenous *RASSF9* gene expression is similar to that of the p21Cip1 expression with a dual pattern in primary keratinocyte differentiation, which was up-regulated by raising calcium to trigger early differentiation, but down-modulated in keratinocytes under terminal differentiation. Furthermore, we suggest that RASSF9 induces p21Cip1 expression in keratinocytes, which are then transitioned from the proliferative compartment to the growth-arrested early differentiation layer, where p21Cip1 functions in early differentiation-related growth arrest (Figure 14A). Conversely, the lack of RASSF9 expression in *RASSF9*−/− mice means that p21Cip1 expression is not sufficiently up-regulated in keratinocytes progressing to early differentiation, allowing these keratinocytes to escape from cell-cycle withdrawal and potentially accounting for the epidermal hyperplasia observed in these mice (Figure 14B).

## Supporting Information

Figure S1 **Specificity of rabbit anti-RASSF9 antiserum.** *RASSF9*−/− primary keratinocytes were transduced with Adv/GFP (GFP) or Adv/HA-RASSF9 (HA-RASSF9) at indicated MOI and incubated in low (−, 0.06 mM) or high (+, 2 mM) calcium ion (Ca^2+^) concentration. Protein lysates of transduced cells were subjected to SDS-PAGE and Western immunoblot using the RASSF9-specific rabbit antiserum generated as described in Materials and Methods at a dilution of 1∶1000. The membrane was reprobed for Keratin-14 (K14) as loading control. In both calcium conditions a major band near 60 kDa, the approximate size of HA-RASSF9, was detected by anti-RASSF9 antiserum only for cells transduced with Adv/HA-RASSF9. The molecular weights of the protein size markers labeled to the right of the blot are, starting at the top, 170, 130, 100, 70, 55, 40, 35, 25, 15 kDa.
(TIF)

Figure S2 **LMP-1 and RASSF9 gene expressions in LMP-1 transgenic mice.** (A) Skin tissue RNA samples of heterozygous and homozygous transgenic mice of 1 day post-partum (dpp; n = 5) and 4 dpp (n = 3) were subjected to RT-PCR for amplification of *LMP-1* and *RASSF9* transcripts. Separately, mouse genotyping was done by PCR of tail DNA using the specific primers for the detection of *LMP-*1 transgene insertion and *RASSF*9 intron deletion. In the cDNA samples of both heterozygotes (1-dpp: Tg-1 and Tg-2; 4-dpp: Tg-6 and Tg-7) and homozygotes (1-dpp: Tg-3 – Tg-5; 4-dpp: Tg-8) no *LMP*-1 transcript was detected. *RASSF9* transcript and the deleted intron sequence targeted by the transgene insertion had been lost in the homozygote samples. For the cDNA samples, NIH-3T3 cells overexpressing full-length LMP-1 and skin tissue of WT mice at 7 dpp were used as positive controls of *LMP-1* and *RASSF9* transcriptions, respectively. Tail genomic DNA extracts of genotyping-confirmed heterozygous mice were used as positive control of presence of both *LMP-*1 transgene insertion (“LMP-1”) and transgene-replaced intron deletion of RASSF9 (“del”). β-Actin was used as internal control. (B) Protein expressions of LMP-1 in LMP-1 transgenic mice at two weeks old were examined by immunoblot with anti-LMP-1 antibody. In both WT mice and the two transgenic mouse lines, Tg1 and Tg2, no LMP-1 protein expression was detected. The asterisk (*) denotes the founder mouse line subsequently used for the experiments reported in this study. 293T cells transfected with LMP-1 expression plasmid constructs pT7(E) and Δ94 [46], [47], and B95-8, the EBV-infected marmoset B-lymphoblastoid cell line [48], were used as positive controls of LMP-1 protein expression. The blot was reprobed for β-Actin as protein loading controls.
(TIF)

Figure S3 **Histological abnormalities in** ***RASSF9*** **−/− skin.** Masson's trichrome staining of skin sections from two-week-old mice. The dashed yellow lines denote the epidermis-dermis and dermis-adipose borders. Similar results were obtained from three independent pairs of mice. The black dashed line-framed areas are enlarged in the panels on the right. Scale bar  = 100 µm. Epi, epidermis; Der, dermis; Adi, adipose.
(TIF)

Figure S4 ***In situ*** **hybridization of** ***RASSF9*** **mRNA in the dorsal skin of mice.** Skin cryosection of two-week-old mice were fixed in paraformaldehyde for in situ hybridization assay. Top right panel: a positive brown signal was developed using an antisense probe against the mRNA for *RASSF9* in WT mice. Top left panel: a sense probe was used as a negative control; no signal was detected in WT mice. Bottom panel: no signal was detected in *RASSF9*−/− mice, irrespective using a sense probe (left) or antisense probe (right), which further confirmed the probe specificity in this system. Similar results were obtained in two independent experiments. Arrow, positive signal in WT epidermis. Scale bar  = 100 µm; +/+, wild type; −/−, *RASSF9*−/−.
(TIF)

Figure S5 **RASSF9 mediates the terminal differentiation of keratinocytes.** (A) The terminal differentiation of *RASSF9*−/− (−/−) versus WT (+/+) mouse keratinocytes was analyzed by immunoblotting with specific antibodies against filaggrin and loricrin. K14 was used as the loading control for keratinocytes. Total cell extracts were prepared from mouse primary keratinocytes incubated for 2 days under growth (0.06 mM calcium) or differentiation-inducing (2 mM calcium) conditions. The intensity of protein expression was determined as the density of the relevant band normalized with respect to that of the K14 loading control; the analysis was performed using the ImageQuant 5.1 software, and the results are shown below the panel. (B) Re-expression of RASSF9 in primary *RASSF9*−/− keratinocytes. Primary *RASSF9*−/− keratinocytes were infected with Adv/HA-*RASSF9* or a GFP-expressing control virus for 24 hr in low-calcium medium, and then switched to fresh growth (0.06 mM calcium) or differentiation-inducing (2 mM calcium) medium for 2 days. The expression levels of the terminal differentiation markers were detected as described in (A). Similar results were obtained in three independent experiments. +/+, wild type; −/−, *RASSF9*−/−.
(TIF)

Figure S6 ***RASSF9*** **−/− mice with a moderate impairment of skin barrier.** Skin permeability assays on *RASSF9*−/− and control newborn pups were performed by toluidine blue dye-penetration assays. *RASSF9*−/− pups showed moderate impaired skin barrier in *RASSF9* deficient pups (−/−), which exhibited slight increase in dye penetration with a punctuated distribution as compared with that of the WT control (+/+). Bottom panel was the enlarged image of *RASSF9*−/− mice marked by * shown in the top panel. Red arrows, signals of dye-penetration; +/+, wild type; +/−, *RASSF9*+/−; −/−; RASSF9 −/−.
(TIF)

Figure S7 **Overexpression of LMP-1 could not reduce the filaggrin expression.** (A) Normal mouse primary keratinocytes were infected with adenoviral vectors encoding LMP-1 or GFP at MOI's = 5 or 10 in low-calcium concentration (0.06 mM) for one-day infection, followed by incubation for 4-day in high-calcium medium (2 mM) as previously described prior to lysis and Western immunoblotting. (B) Intensity measurements of the filaggrin bands. Data represent two separate blots. No statistical differences in band intensities were detected.
(TIF)

Figure S8 **Histological abnormalities in developing** ***RASSF9*** **−/− lung.** H&E staining of lung sections of mice at two weeks old. The *RASSF9*−/− lung (−/−) exhibits stunted progress of saccule septation versus WT lung (+/+), with grossly enlarged alveoli. In the panels of *RASSF9*−/− section noticeable thickening of pulmonary alveolar epithelium can be observed (asterisks, *). (A) Low-magnification views of pulmonary alveoli. Scale bar  = 200 µm; (B,C) High-magnification views of pulmonary alveoli. Scale bar  = 40 µm.
(TIF)

Figure S9 **Decrease of p21Cip1 (** ***CDKN1A*** **) mRNA in** ***RASSF9*** **−/− skin.** Total RNA of mice skins were extracted by TRIzol reagent, and used to determine the mRNA level of p21Cip1 in mouse skin tissue by QRT-PCR using p21Cip1 gene-specific primers followed by normalization with regard to the mRNA expression of the reference gene: (A) β-Actin, (B) *GAPDH* and (C) *HPRT1*. Mean ± SD (n = 3, per genotypes); *, *P*<0.05; +/+, wild type; −/−, *RASSF9*−/−.
(TIF)

Figure S10 **Expression profile of** ***RASSF9*** **mRNA in normal mouse primary keratinocytes.** Total RNA was prepared from mouse primary keratinocytes cultured for the indicated times in growth medium (0.06 mM calcium) or differentiation-inducing medium (2 mM calcium). *RASSF9* mRNA expression was determined by QRT-PCR analysis using *RASSF9* gene-specific primers followed by normalization with regard to the mRNA expression of the reference gene: (A) β-Actin, (B) *GAPDH* and (C) *HPRT1*. The results shown are given as the fold-change (Mean ± SD, n = 3); * *P*<0.05; ** *P*<0.005.
(TIF)

Figure S11 ***RASSF9+/*** **− keratinocytes could be induced to differentiate in a manner similar to that of WT keratinocytes.** keratinocytes from *RASSF9*-deficient heterozygotes or WT mice were cultured under growth (0.06 mM calcium) or differentiation-inducing (2 mM calcium) conditions for 4 days, and the expression levels of filaggrin (a marker for terminal differentiation) and K14 (loading control) were analyzed by Western blotting. Similar results were obtained in three independent experiments. +/+, wild type; +/−, *RASSF9*+/−.
(TIF)

Figure S12 **RASSF9 expression could overcome the differentiation defects in** ***RASSF9*** **−/− keratinocytes, but increasing RASSF9 expression did not enhance terminal differentiation in keratinocytes.** (A) *RASSF9*−/− mouse primary keratinocytes were transduced by adenoviral vectors encoding HA-tagged RASSF9 or the GFP control with a viral dose of 5, 10 and 20 MOI for 1-day infection, and incubated in differentiation-inducing medium (2 mM calcium) for 4 days. Western blotting was used to examine the expression levels of filaggrin (a terminal differentiation marker) and HA-RASSF9. Similar results were obtained in two independent experiments. (B) Normal mouse primary keratinocytes were infected with adenoviral vectors encoding HA-tagged RASSF9 or the GFP control with a viral dose of 5, 10, 15 and 20 MOI for 1-day infection, then incubated as described in (A) before lysis and Western blot analysis. +/+, wild type; −/−, *RASSF9*−/−.
(TIF)

Table S1 **Oligonucleotide primers used to amplify the fragments for Southern probes, transgene-flanking genome and gene identification.**
(DOC)
