# Supplementary figures and images for: A Novel Role of RASSF9 in Maintaining Epidermal Homeostasis
Source: PLoS One. 2011 Mar 21;6(3):e17867. doi: 10.1371/journal.pone.0017867 (PMC3061870; doi:10.1371/journal.pone.0017867)

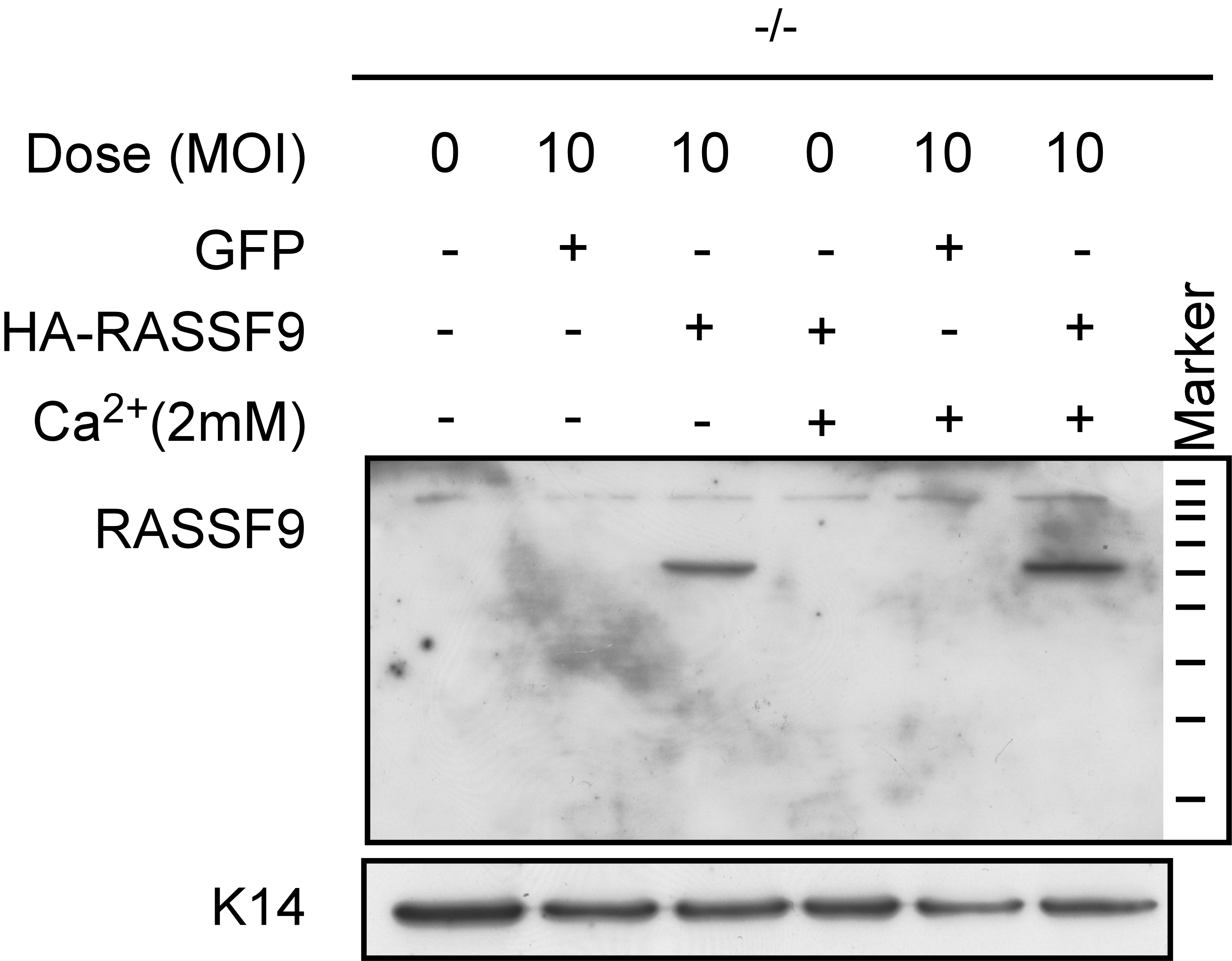

Supplement: Figure S1 — Specificity of rabbit anti-RASSF9 antiserum. RASSF9−/− primary keratinocytes were transduced with Adv/GFP (GFP) or Adv/HA-RASSF9 (HA-RASSF9) at indicated MOI and incubated in low (−, 0.06 mM) or high (+, 2 mM) calcium ion (Ca2+) concentration. Protein lysates of transduced cells were subjected to SDS-PAGE and Western immunoblot using the RASSF9-specific rabbit antiserum generated as described in Materials and Methods at a dilution of 1∶1000. The membrane was reprobed for Keratin-14 (K14) as loading control. In both calcium conditions a major band near 60 kDa, the approximate size of HA-RASSF9, was detected by anti-RASSF9 antiserum only for cells transduced with Adv/HA-RASSF9. The molecular weights of the protein size markers labeled to the right of the blot are, starting at the top, 170, 130, 100, 70, 55, 40, 35, 25, 15 kDa. (TIF) [file pone.0017867.s001.tif]

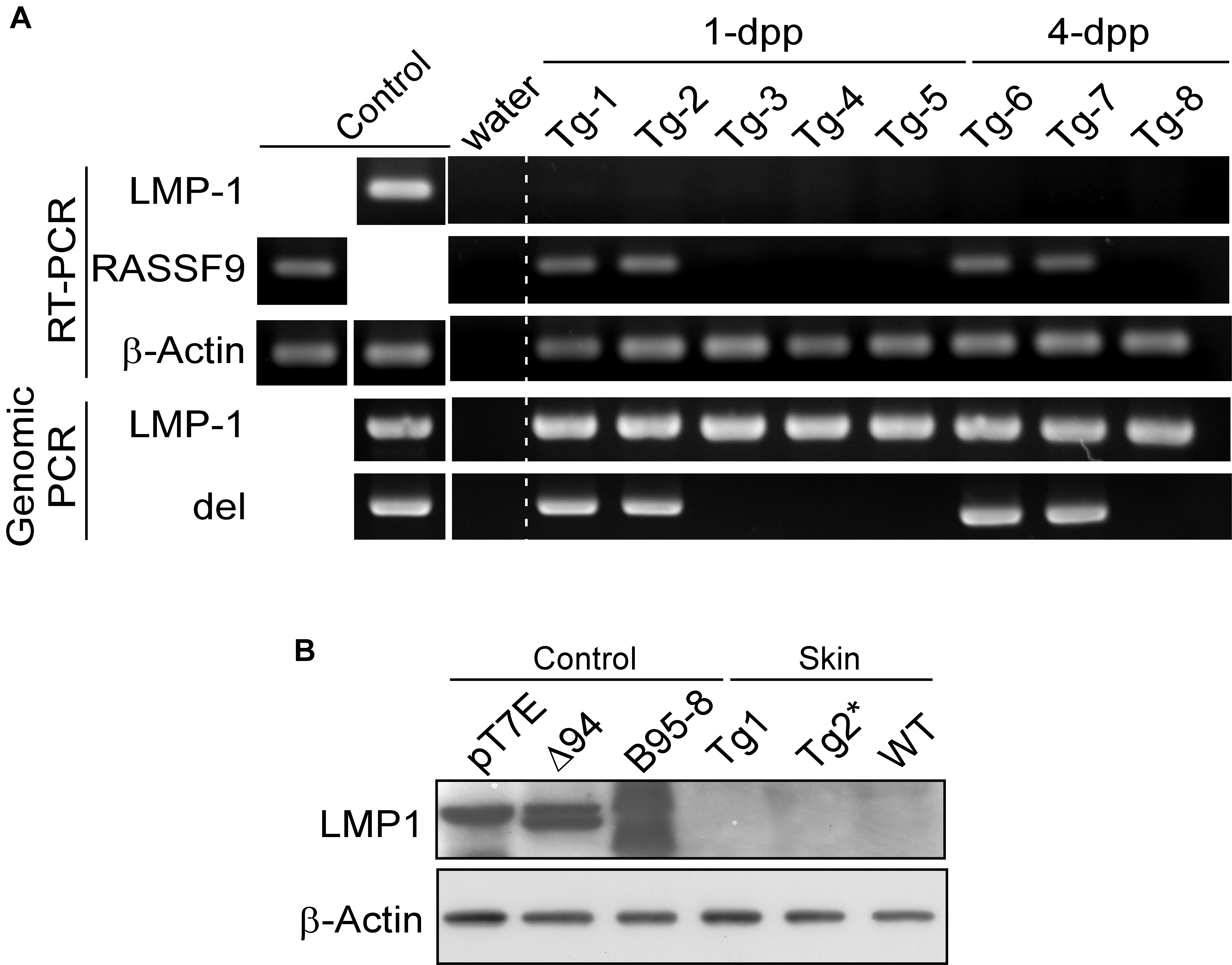

Supplement: Figure S2 — LMP-1 and RASSF9 gene expressions in LMP-1 transgenic mice. (A) Skin tissue RNA samples of heterozygous and homozygous transgenic mice of 1 day post-partum (dpp; n = 5) and 4 dpp (n = 3) were subjected to RT-PCR for amplification of LMP-1 and RASSF9 transcripts. Separately, mouse genotyping was done by PCR of tail DNA using the specific primers for the detection of LMP-1 transgene insertion and RASSF9 intron deletion. In the cDNA samples of both heterozygotes (1-dpp: Tg-1 and Tg-2; 4-dpp: Tg-6 and Tg-7) and homozygotes (1-dpp: Tg-3 – Tg-5; 4-dpp: Tg-8) no LMP-1 transcript was detected. RASSF9 transcript and the deleted intron sequence targeted by the transgene insertion had been lost in the homozygote samples. For the cDNA samples, NIH-3T3 cells overexpressing full-length LMP-1 and skin tissue of WT mice at 7 dpp were used as positive controls of LMP-1 and RASSF9 transcriptions, respectively. Tail genomic DNA extracts of genotyping-confirmed heterozygous mice were used as positive control of presence of both LMP-1 transgene insertion (“LMP-1”) and transgene-replaced intron deletion of RASSF9 (“del”). β-Actin was used as internal control. (B) Protein expressions of LMP-1 in LMP-1 transgenic mice at two weeks old were examined by immunoblot with anti-LMP-1 antibody. In both WT mice and the two transgenic mouse lines, Tg1 and Tg2, no LMP-1 protein expression was detected. The asterisk (*) denotes the founder mouse line subsequently used for the experiments reported in this study. 293T cells transfected with LMP-1 expression plasmid constructs pT7(E) and Δ94 [46], [47], and B95-8, the EBV-infected marmoset B-lymphoblastoid cell line [48], were used as positive controls of LMP-1 protein expression. The blot was reprobed for β-Actin as protein loading controls. (TIF) [file pone.0017867.s002.tif]

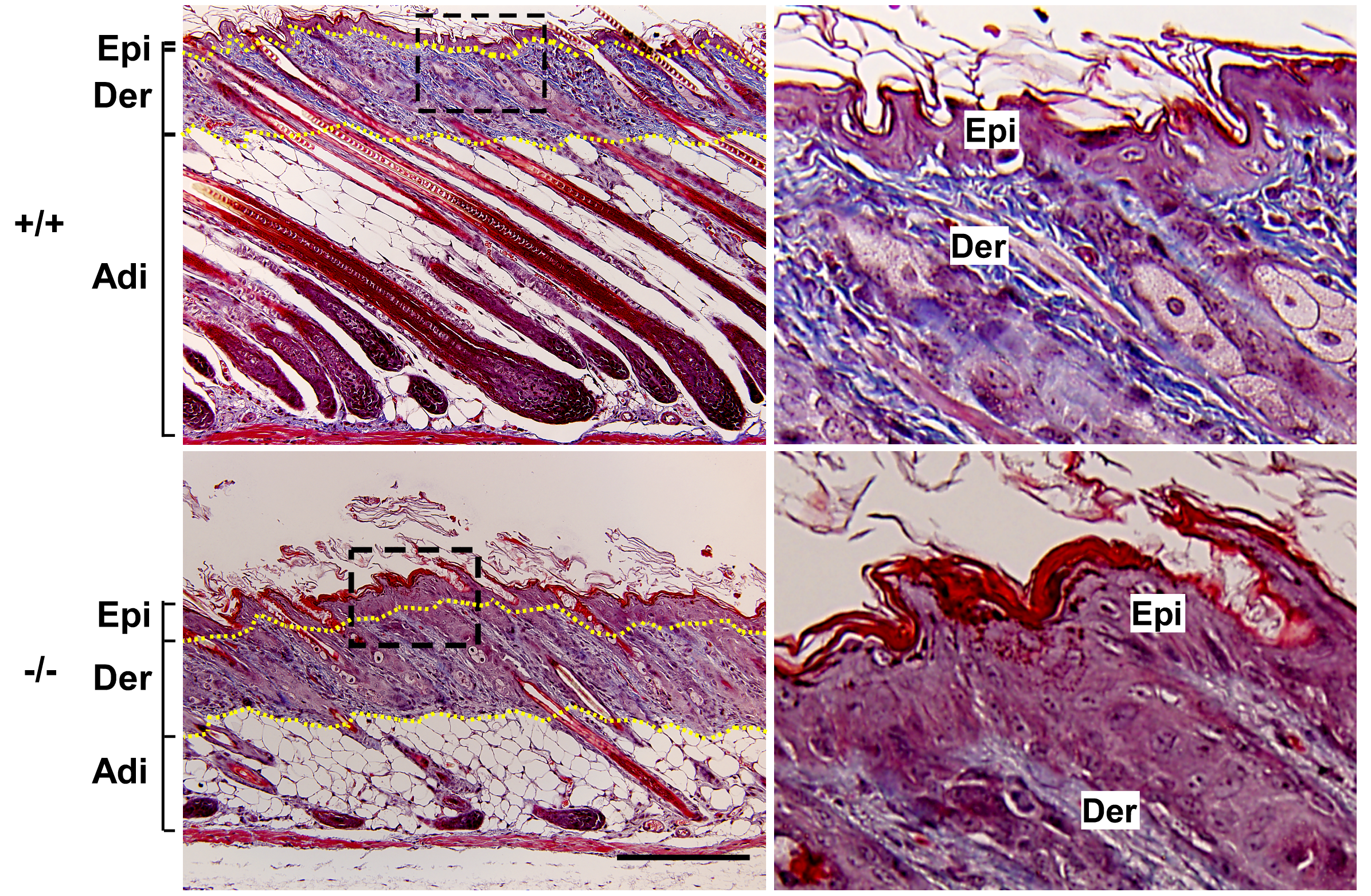

Supplement: Figure S3 — Histological abnormalities in RASSF9 −/− skin. Masson's trichrome staining of skin sections from two-week-old mice. The dashed yellow lines denote the epidermis-dermis and dermis-adipose borders. Similar results were obtained from three independent pairs of mice. The black dashed line-framed areas are enlarged in the panels on the right. Scale bar = 100 µm. Epi, epidermis; Der, dermis; Adi, adipose. (TIF) [file pone.0017867.s003.tif]

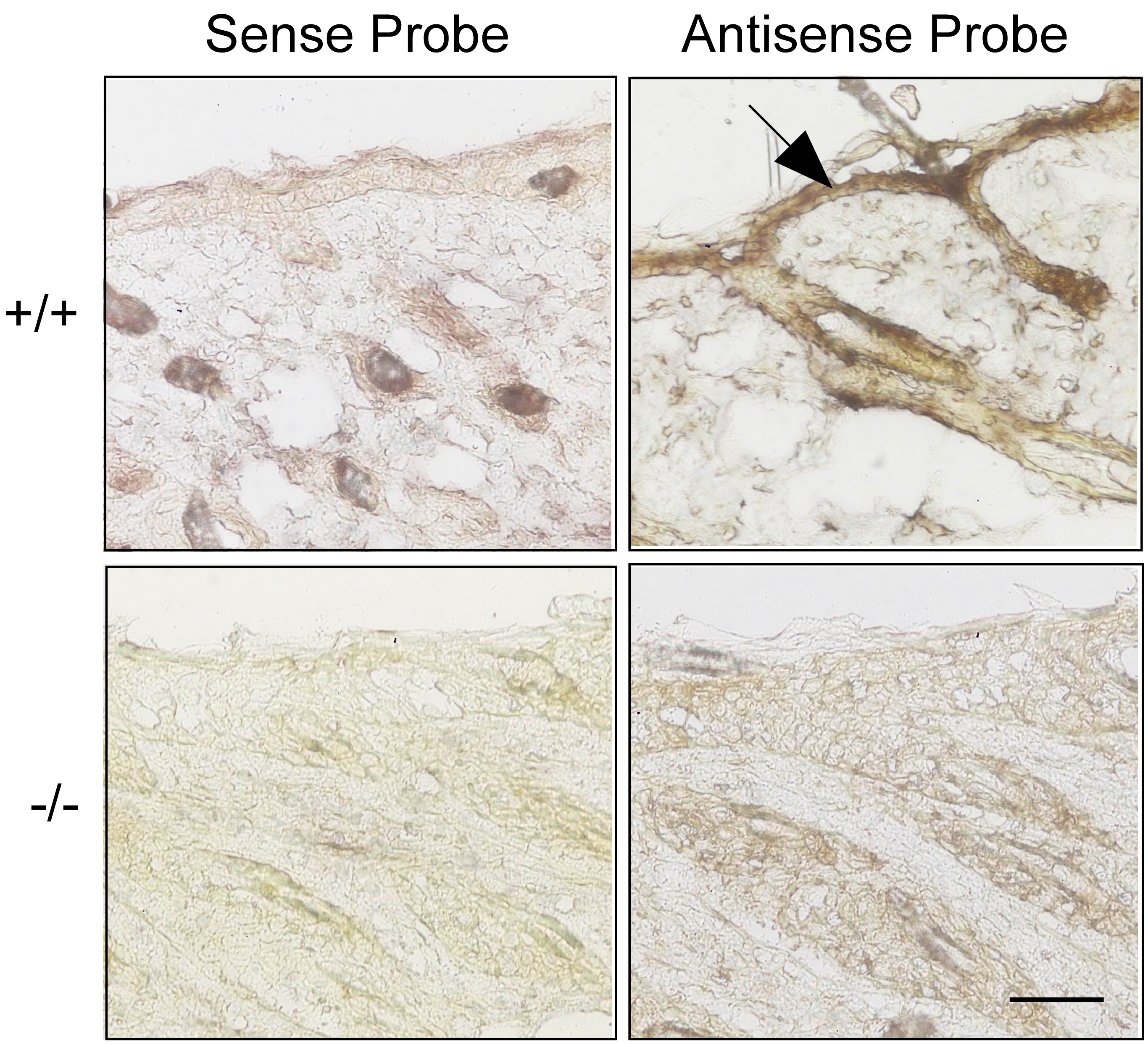

Supplement: Figure S4 — In situ hybridization of RASSF9 mRNA in the dorsal skin of mice. Skin cryosection of two-week-old mice were fixed in paraformaldehyde for in situ hybridization assay. Top right panel: a positive brown signal was developed using an antisense probe against the mRNA for RASSF9 in WT mice. Top left panel: a sense probe was used as a negative control; no signal was detected in WT mice. Bottom panel: no signal was detected in RASSF9−/− mice, irrespective using a sense probe (left) or antisense probe (right), which further confirmed the probe specificity in this system. Similar results were obtained in two independent experiments. Arrow, positive signal in WT epidermis. Scale bar = 100 µm; +/+, wild type; −/−, RASSF9−/−. (TIF) [file pone.0017867.s004.tif]

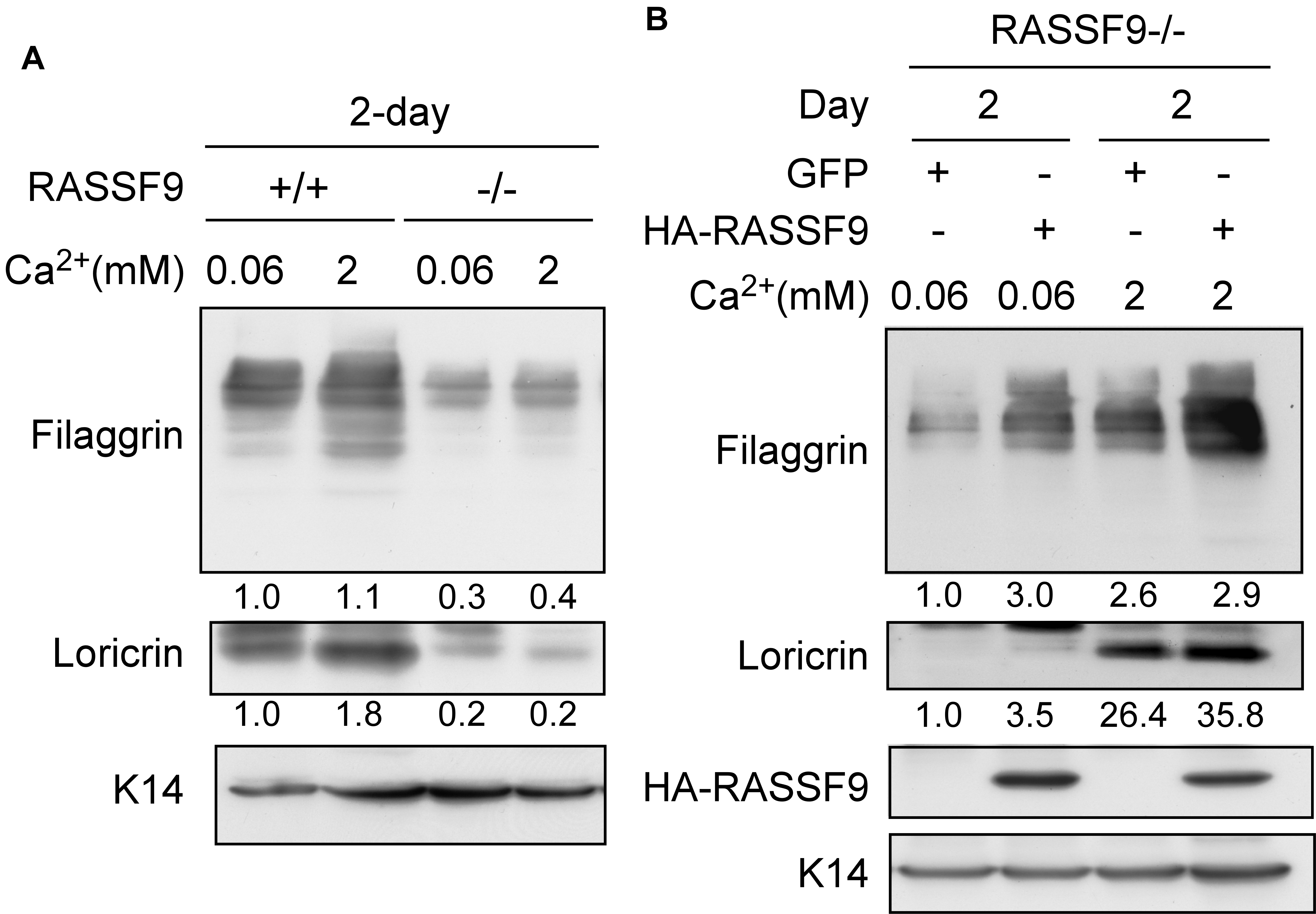

Supplement: Figure S5 — RASSF9 mediates the terminal differentiation of keratinocytes. (A) The terminal differentiation of RASSF9−/− (−/−) versus WT (+/+) mouse keratinocytes was analyzed by immunoblotting with specific antibodies against filaggrin and loricrin. K14 was used as the loading control for keratinocytes. Total cell extracts were prepared from mouse primary keratinocytes incubated for 2 days under growth (0.06 mM calcium) or differentiation-inducing (2 mM calcium) conditions. The intensity of protein expression was determined as the density of the relevant band normalized with respect to that of the K14 loading control; the analysis was performed using the ImageQuant 5.1 software, and the results are shown below the panel. (B) Re-expression of RASSF9 in primary RASSF9−/− keratinocytes. Primary RASSF9−/− keratinocytes were infected with Adv/HA-RASSF9 or a GFP-expressing control virus for 24 hr in low-calcium medium, and then switched to fresh growth (0.06 mM calcium) or differentiation-inducing (2 mM calcium) medium for 2 days. The expression levels of the terminal differentiation markers were detected as described in (A). Similar results were obtained in three independent experiments. +/+, wild type; −/−, RASSF9−/−. (TIF) [file pone.0017867.s005.tif]

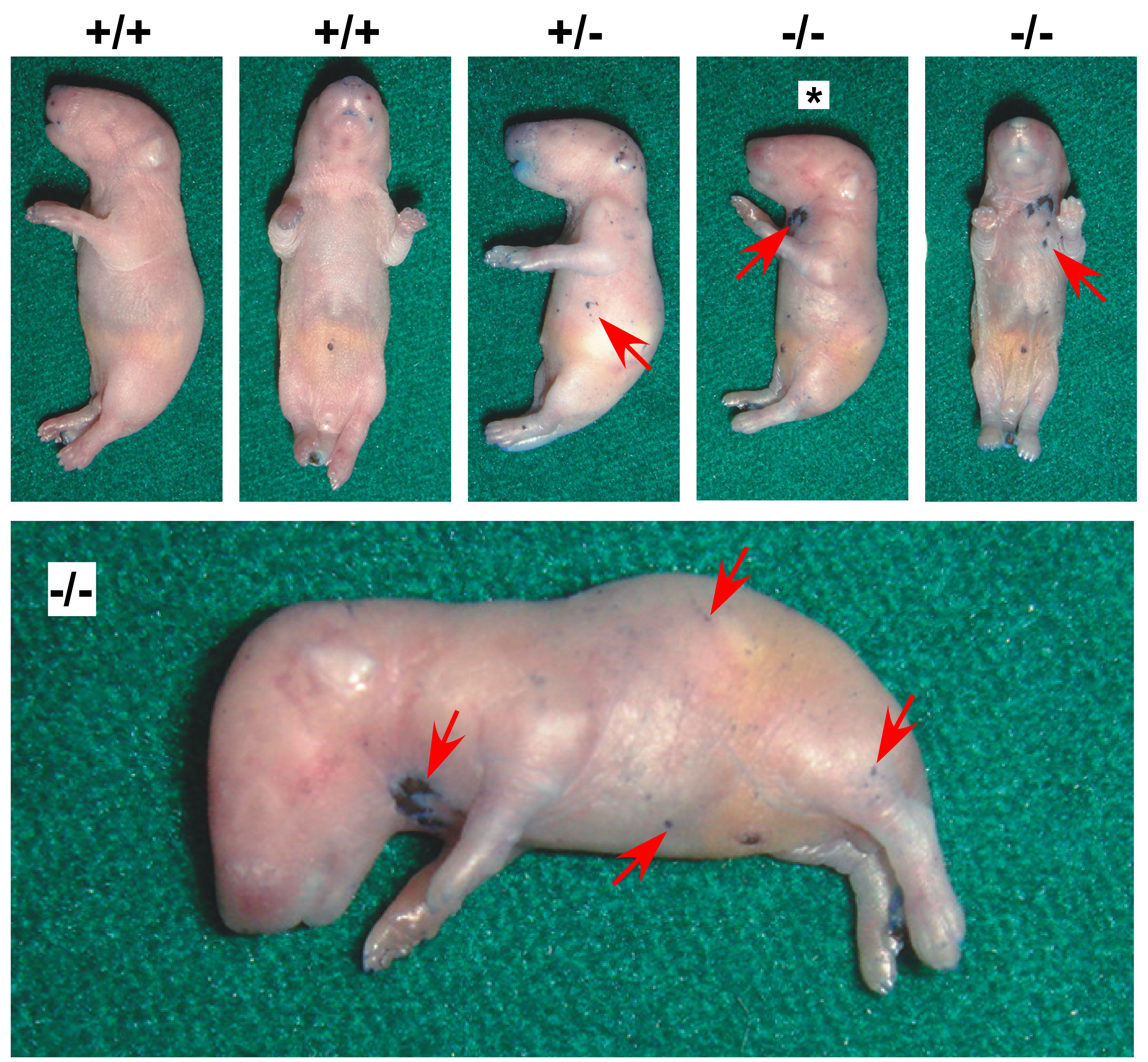

Supplement: Figure S6 — RASSF9 −/− mice with a moderate impairment of skin barrier. Skin permeability assays on RASSF9−/− and control newborn pups were performed by toluidine blue dye-penetration assays. RASSF9−/− pups showed moderate impaired skin barrier in RASSF9 deficient pups (−/−), which exhibited slight increase in dye penetration with a punctuated distribution as compared with that of the WT control (+/+). Bottom panel was the enlarged image of RASSF9−/− mice marked by * shown in the top panel. Red arrows, signals of dye-penetration; +/+, wild type; +/−, RASSF9+/−; −/−; RASSF9 −/−. (TIF) [file pone.0017867.s006.tif]

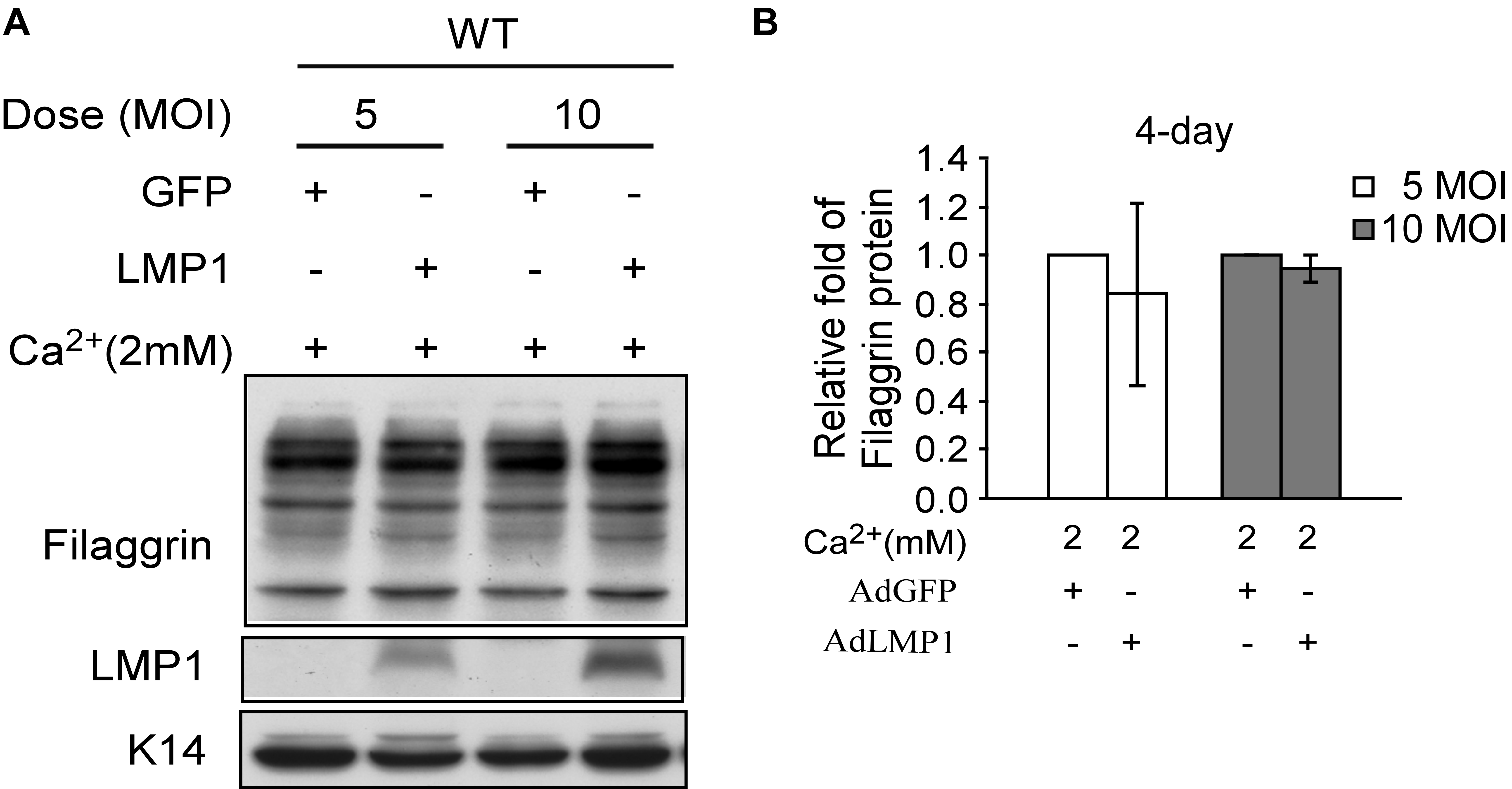

Supplement: Figure S7 — Overexpression of LMP-1 could not reduce the filaggrin expression. (A) Normal mouse primary keratinocytes were infected with adenoviral vectors encoding LMP-1 or GFP at MOI's = 5 or 10 in low-calcium concentration (0.06 mM) for one-day infection, followed by incubation for 4-day in high-calcium medium (2 mM) as previously described prior to lysis and Western immunoblotting. (B) Intensity measurements of the filaggrin bands. Data represent two separate blots. No statistical differences in band intensities were detected. (TIF) [file pone.0017867.s007.tif]

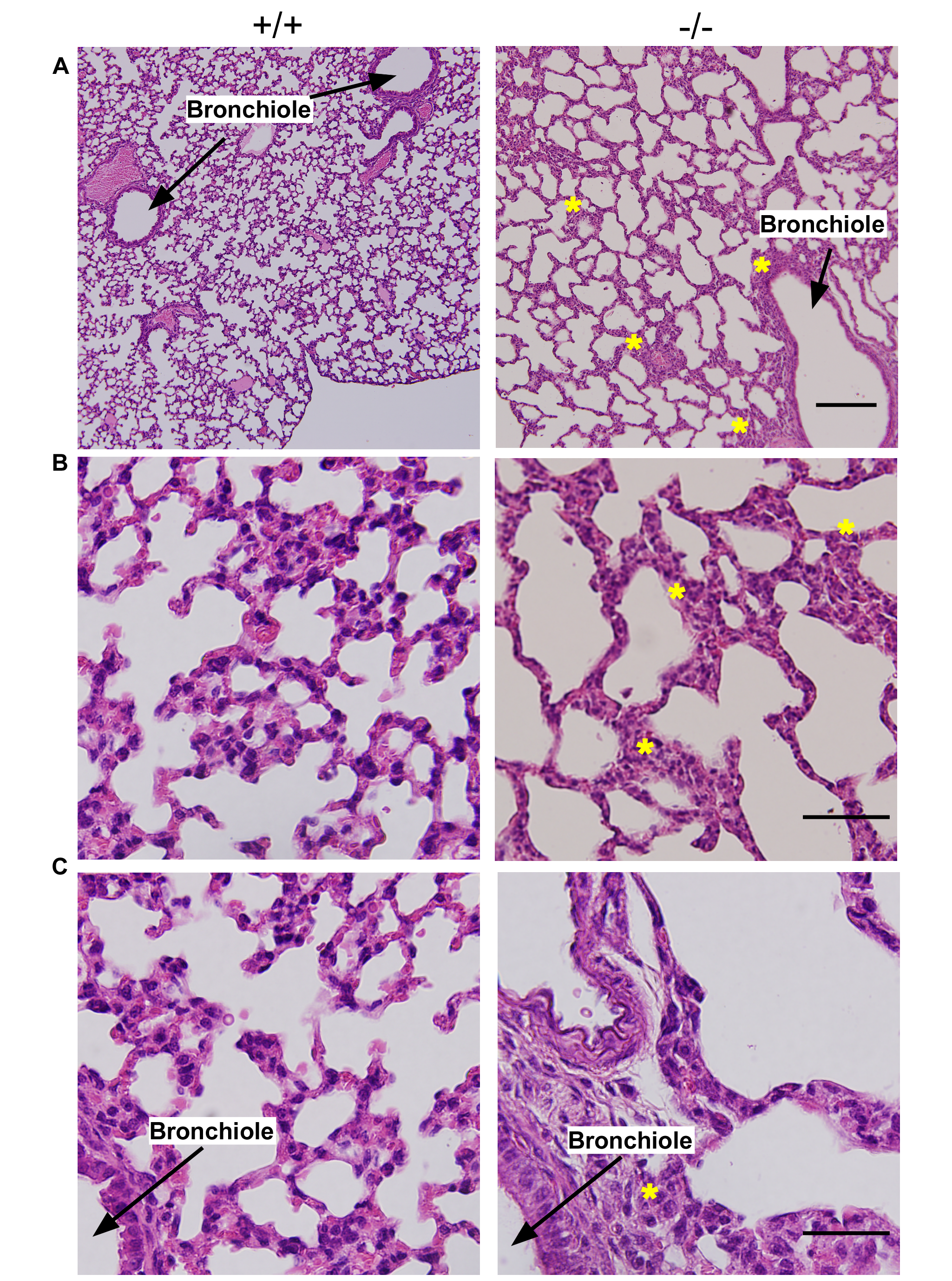

Supplement: Figure S8 — Histological abnormalities in developing RASSF9 −/− lung. H&E staining of lung sections of mice at two weeks old. The RASSF9−/− lung (−/−) exhibits stunted progress of saccule septation versus WT lung (+/+), with grossly enlarged alveoli. In the panels of RASSF9−/− section noticeable thickening of pulmonary alveolar epithelium can be observed (asterisks, *). (A) Low-magnification views of pulmonary alveoli. Scale bar = 200 µm; (B,C) High-magnification views of pulmonary alveoli. Scale bar = 40 µm. (TIF) [file pone.0017867.s008.tif]

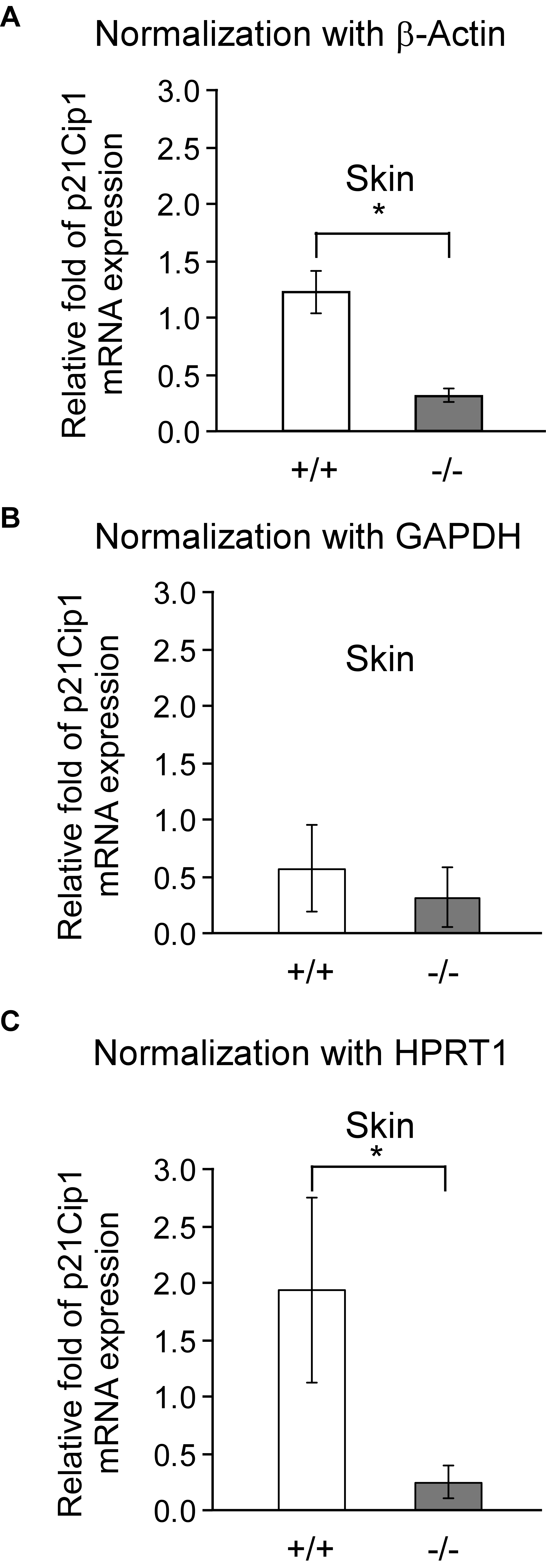

Supplement: Figure S9 — Decrease of p21Cip1 ( CDKN1A ) mRNA in RASSF9 −/− skin. Total RNA of mice skins were extracted by TRIzol reagent, and used to determine the mRNA level of p21Cip1 in mouse skin tissue by QRT-PCR using p21Cip1 gene-specific primers followed by normalization with regard to the mRNA expression of the reference gene: (A) β-Actin, (B) GAPDH and (C) HPRT1. Mean ± SD (n = 3, per genotypes); *, P<0.05; +/+, wild type; −/−, RASSF9−/−. (TIF) [file pone.0017867.s009.tif]

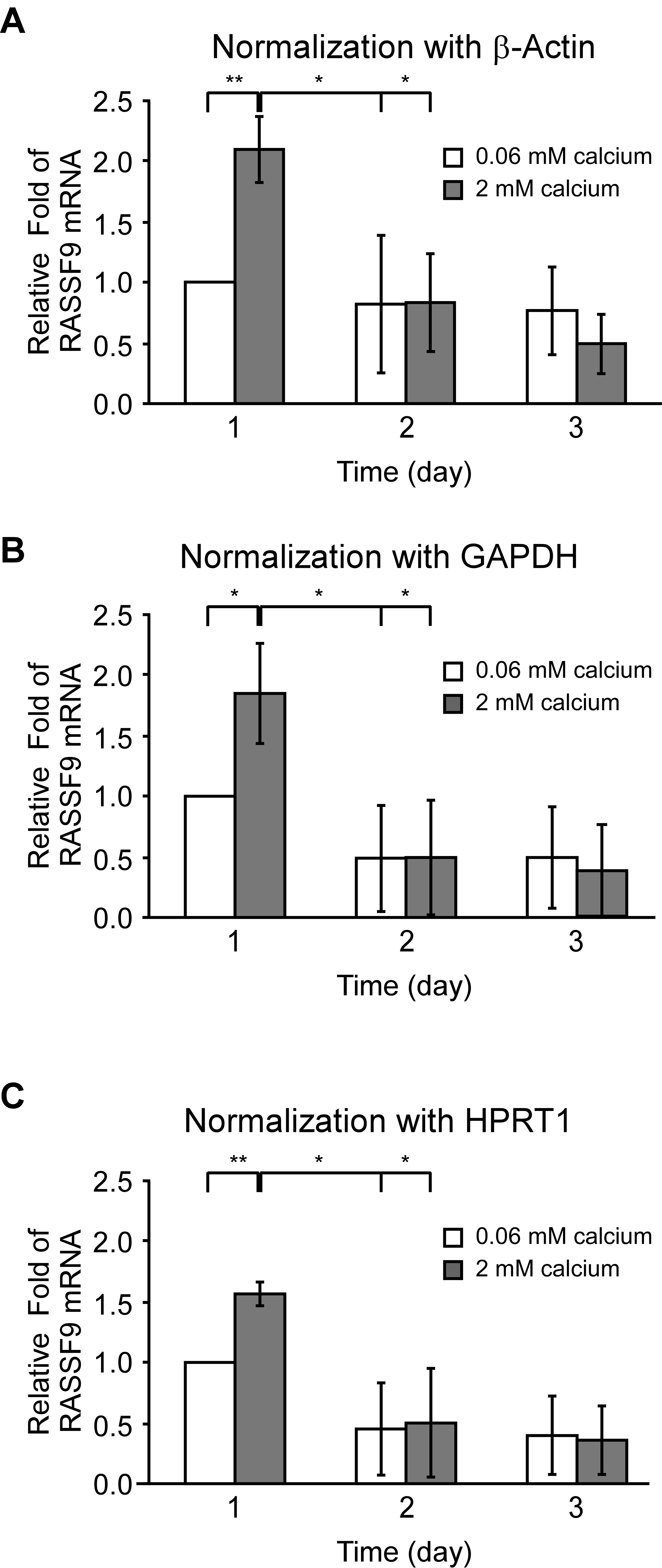

Supplement: Figure S10 — Expression profile of RASSF9 mRNA in normal mouse primary keratinocytes. Total RNA was prepared from mouse primary keratinocytes cultured for the indicated times in growth medium (0.06 mM calcium) or differentiation-inducing medium (2 mM calcium). RASSF9 mRNA expression was determined by QRT-PCR analysis using RASSF9 gene-specific primers followed by normalization with regard to the mRNA expression of the reference gene: (A) β-Actin, (B) GAPDH and (C) HPRT1. The results shown are given as the fold-change (Mean ± SD, n = 3); * P<0.05; ** P<0.005. (TIF) [file pone.0017867.s010.tif]

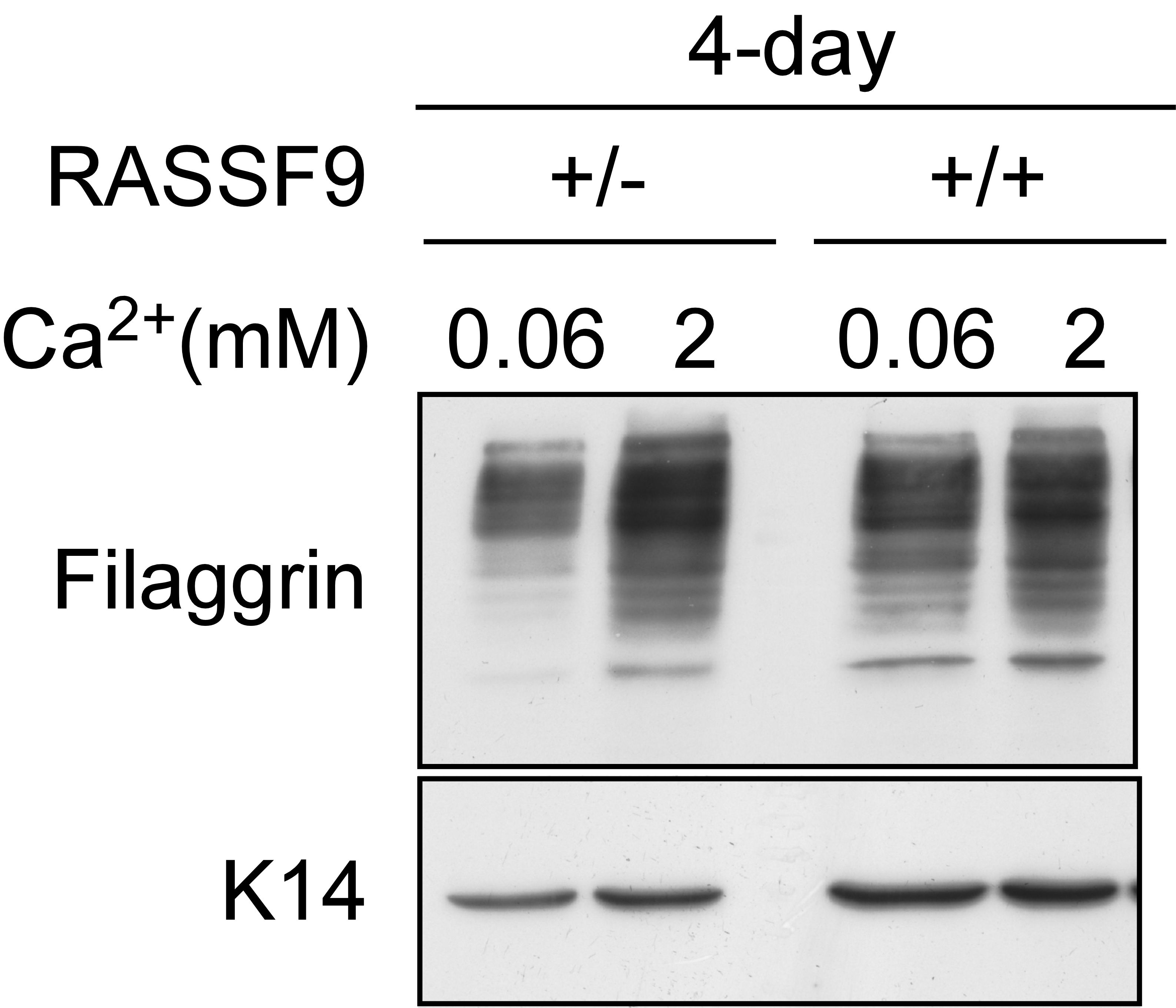

Supplement: Figure S11 — RASSF9+/ − keratinocytes could be induced to differentiate in a manner similar to that of WT keratinocytes. keratinocytes from RASSF9-deficient heterozygotes or WT mice were cultured under growth (0.06 mM calcium) or differentiation-inducing (2 mM calcium) conditions for 4 days, and the expression levels of filaggrin (a marker for terminal differentiation) and K14 (loading control) were analyzed by Western blotting. Similar results were obtained in three independent experiments. +/+, wild type; +/−, RASSF9+/−. (TIF) [file pone.0017867.s011.tif]

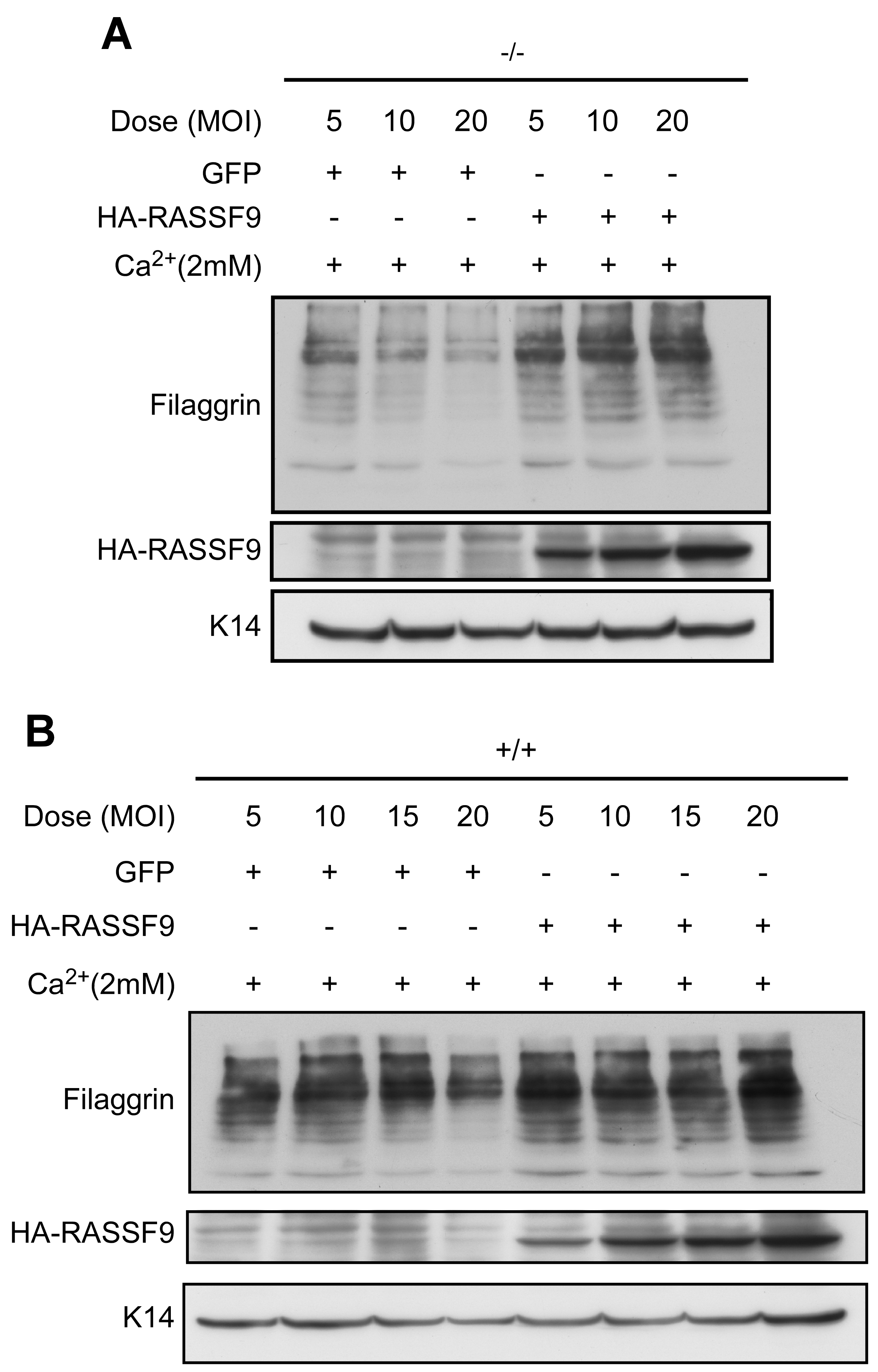

Supplement: Figure S12 — RASSF9 expression could overcome the differentiation defects in RASSF9 −/− keratinocytes, but increasing RASSF9 expression did not enhance terminal differentiation in keratinocytes. (A) RASSF9−/− mouse primary keratinocytes were transduced by adenoviral vectors encoding HA-tagged RASSF9 or the GFP control with a viral dose of 5, 10 and 20 MOI for 1-day infection, and incubated in differentiation-inducing medium (2 mM calcium) for 4 days. Western blotting was used to examine the expression levels of filaggrin (a terminal differentiation marker) and HA-RASSF9. Similar results were obtained in two independent experiments. (B) Normal mouse primary keratinocytes were infected with adenoviral vectors encoding HA-tagged RASSF9 or the GFP control with a viral dose of 5, 10, 15 and 20 MOI for 1-day infection, then incubated as described in (A) before lysis and Western blot analysis. +/+, wild type; −/−, RASSF9−/−. (TIF) [file pone.0017867.s012.tif]
