# Supplementary material for: A Novel Role of RASSF9 in Maintaining Epidermal Homeostasis
Source: PLoS One. 2011 Mar 21;6(3):e17867. doi: 10.1371/journal.pone.0017867 (PMC3061870; doi:10.1371/journal.pone.0017867)
Supplement: Table S1 — Oligonucleotide primers used to amplify the fragments for Southern probes, transgene-flanking genome and gene identification. (DOC) [file pone.0017867.s013.doc]

**Table S1.** Oligonucleotide primers used to amplify the fragments for Southern probes, transgene-flanking genome and gene identification.

| **Name** | primer (5’-3’) | Purpose |
| --- | --- | --- |
| **Δ_probe** | ATGGTTGGCCCATTAATAGC | Southern probe |
|  | CAGGCCTAGAAGGAATGTAG |  |
| **Tg_peobe** | ACAATGCCTGTCCGTGC | Southern probe |
|  | AGAAACACGCGTTACTCT |  |
| **RI** | TTGCTCCTGCCACACTACCCTGACC | Inverse PCR of RIF |
|  | GAGTAACTGGACTGGAGGAGCGCTCC |  |
| **H3** | ACAGGACCTATGGTCTGAAGAGCTAGCTGG | Inverse PCR of H3F |
|  | CACGACACACTGATGAACACCACCACGATG |  |
| **Δ_PCR** | CGTGTAACAGGGGTTAGCAATTGC  GGGTGTTGGAGTTGTAGGTACAAG | Genotype screening; primers specific for the deleted sequence of the RASSF9 intron and *LMP*-1 transgene |
| ***LMP*1_PCR1** | GGTTGATCTCCTTTGGCTCCTCCTG  AGTCAGTCAGGCAAGCCTATGACATG |  |
| ***LMP*1_PCR2** | CCTTCTCTGTCCACTTGG  GGATCGGAAGAAGGATCGGT | RT-PCR |
| ***RASSF9*** | GACCATGGCTCCCTTTGGA | QRT-PCR and PCR |
|  | TGGAGGTGGTGCGTTTAGTTAA |  |
| ***CDKN1A*** | CAGACATTCAGAGCCACAGG | QRT-PCR |
|  | TCAAAGTTCCACCGTTCTCG |  |
| ***CDH1*** | AGGAAATCACATCTTATACCGC | QRT-PCR |
|  | CGTCTTCTCTGTCCATCTCA |  |
| ***fn1*** | AGGCAGAAAACAGGTCTCGATT | QRT-PCR |
|  | CAGAATGCTCGGCGTGATG |  |
| **β-Actin** | CATTGCTGACAGGATGCAGAA | QRT-PCR and PCR |
|  | GCTGATCCACATCTGCTGGAA |  |
| **GAPDH** | ATCACTGCCACCCAGAAGAC  CAGTGAGCTTCCCGTTCAG | QRT-PCR |
| **HPRT1** | TGGGCTTACCTCACTGCTTTCC  CCTGGTTCATCATCGCTAATCACG | QRT-PCR |
